# Lipoprotein subclasses and gastrointestinal cancers: novel perspectives and potential associations

**DOI:** 10.3389/fnut.2025.1501263

**Published:** 2025-03-03

**Authors:** Chuang Yang, Dong Liu, Yong Wang, Feng Cao

**Affiliations:** ^1^Medical Faculty, University of Leipzig, Leipzig, Germany; ^2^Department of General, Visceral and Transplantation Surgery, University Hospital RWTH Aachen, Aachen, Germany; ^3^Department of General Surgery, The Shenzhen Hospital of Southern Medical University, Shenzhen, China; ^4^Medical Faculty, RWTH Aachen University, Aachen, Germany

**Keywords:** lipoprotein subclasses, gastrointestinal (GI) cancers, UK Biobank (UKB), Mendelian randomization (MR), metabolomics

## Abstract

**Background:**

This study aimed to investigate the associations between serum lipoprotein subclasses and the long-term risk of gastrointestinal (GI) cancers to enhance our understanding of the etiology of GI cancers.

**Methods:**

This prospective cohort study included 249,450 participants from the UK Biobank. Cox proportional hazard models were used to assess the association between 17 serum lipoprotein subclasses with the risk of GI cancers. Restricted cubic spline (RCS) analysis was employed to assess the corresponding dose–response relationships. Additionally, Mendelian randomization (MR) analysis was used to evaluate the causal relationships between the lipoproteins and the risk of GI cancers.

**Results:**

A total of 4,787 cases of GI cancers were recorded over a median follow-up period of 12.92 years. Our results revealed that the majority of the high-density lipoprotein (HDL) subclasses, such as very large-, large-, and medium-HDL-particles, were positively associated, while several low-density lipoprotein (LDL) subclasses were negatively associated with the risk of overall GI cancer. Additionally, RCS analysis revealed a linear dose–response relationship between elevated levels of most lipoprotein particles and the risk of overall GI cancer development. Additionally, subgroup analysis indicated a significant sex-dependent interaction between lipoprotein particles and the risk of GI cancers. However, MR analysis revealed a different causal relationships between lipoprotein and GI cancers at the genetic level.

**Conclusion:**

In this large-scale metabolomics study, we identified several associations between lipoprotein subclasses and the long-term risk of GI cancers. However, further research is needed to fully elucidate their roles in the mechanisms of cancer development.

## Introduction

Gastrointestinal (GI) cancers are a prevalent and severe category of diseases that pose a significant challenge to global health. GI cancers encompass various types of cancers, including esophageal cancer (EC), gastric cancer (GC), liver cancer (LC), and colorectal cancer (CRC) (1, 2). GI cancers have the highest incidence rate among malignant tumors, with nearly 5 million new cases of GI cancers reported worldwide in 2022, accounting for approximately one-fourth of all new cancer cases (3, 4). Despite several advances in cancer research over the past decades, research on the etiology and treatment modalities for GI cancers remains limited.

In recent years, lipoproteins have emerged as key players in the onset and progression of diseases. They not only participate in cholesterol metabolism and transport but also exhibit close associations with various physiological and pathological processes, such as inflammation and immune regulation (5, 6). Moreover, aberrant changes in lipoproteins were found to be associated with tumor initiation and development, gaining widespread attention. For instance, Revilla et al. (7) and Sun et al. (8) demonstrated that high/low-density lipoproteins (HDL/LDL) regulate the progression of endocrine-related tumors. Additionally, Samadi et al. (9) suggested that the reverse cholesterol transport function of HDL may serve as a potential therapeutic target for BC. Ganjali et al. (10) proposed an association between LDL levels and increased cancer risk. In contrast, some clinical studies found that statin drugs, which lower cholesterol levels, do not exhibit any significant correlation with cancer incidence or mortality (11, 12). However, our understanding of the biological functions of lipoproteins in GI cancers remains limited. Current controversies in the role of lipoproteins in GI cancers may stem from the simplistic view of lipoproteins and the lack of their differentiation based on size, concentration, and composition. The development of nuclear magnetic resonance (NMR) techniques enabled the quantitative measurement of lipoprotein particles and their size distribution. Increasing evidence from NMR studies suggests that lipoprotein particles are heterogeneous in size and biochemical composition, with different subgroups potentially possessing distinct functional characteristics (13).

This study aims to explore the specific functions of different lipoprotein subclasses and their association with GI cancers at both clinical and genetic levels. A thorough understanding of the functionality and potential of various lipoprotein subclasses in gastrointestinal cancer will enable the development of therapeutic strategies tailored to these subclasses, further enhancing treatment efficacy and patient survival rates.

## Methods

### Data source

The UK Biobank (UKB), a multi-center large-scale biomedical cohort study, was conducted in the United Kingdom during 2006–2010 and consisted of over 500,000 participants aged 37–73 years old. The detailed methodology of the project has been comprehensively outlined in a previous publication (14). The research project was approved by the Northwest Multicenter Research Ethics Committee, and all the recruited participants actively engaged and consented to the study. This study adhered to the reporting standards outlined in the Strengthening the Reporting of Observational Studies in Epidemiology (STROBE) guideline (STROBE-MR Checklist).

### Measurement of lipoprotein data

Nightingale Health utilized a high-throughput NMR metabolomics platform to conduct a metabolomic analysis of the baseline plasma samples of over 270,000 participants in the UKB between 2019 and 2022 (15). A total of 249 metabolites, including lipids, fatty acids, amino acids and other low-molecular-weight metabolites, were assessed in this study, along with the analysis of the distribution, particle size, and composition of lipoprotein subclasses (16). For this study, we obtained data of 17 lipoprotein subclasses from NMR metabolomics platform.

### Verification of outcomes

The primary endpoint of the study was the overall risk of GI cancer, and the secondary endpoint of the study was the risk of each specific GI cancer, including EC, GC, small intestine cancer (SIC), CRC, LC, and pancreatic cancer (PC). The diagnoses of GI cancer were based on the International Classification of Diseases, Tenth Revision (ICD-10) codes (Supplementary file 1: eTable 1). The subtypes of EC [esophageal adenocarcinoma (EAC), esophageal squamous cell carcinoma (ESCC)] and LC [hepatocellular carcinoma (HCC), cholangiocarcinoma (CAC)] were also included (Supplementary file 1: eTable 2). The last follow-up date was defined as the date of the last occurrence of cancer cases (June 1, 2022). The follow-up period for each participant started from their enrollment date and continued until the first occurrence of the aforementioned GI cancers, death, or censoring, whichever comes first.

### Assessment of other variables

The baseline sociodemographic characteristics of the patients, including age, sex, body mass index (BMI), ethnicity, Townsend deprivation index (TDI), alcohol and smoking status, dietary habits, history of chronic diseases, and medication usage, were collected via touchscreen data collection or brief interviews. The TDI reflects the socioeconomic status of the participants (17). Physical activity levels were assessed using weekly metabolic equivalent (MET) minutes (18). The diet score was based on the consumption of nine food items, namely processed meat, red meat, fish, milk, spreads, cereals, added salt, water intake, and fruits and vegetables (Supplementary file 1: eTable 3) (19).

### Selection criteria

Participants who were diagnosed with any type of cancer at recruitment (*n* = 45,777), lacked entry time information (*n* = 2), or lacked lipoprotein subclass data (*n* = 207,128) were excluded from this study. Ultimately, a total of 249,450 participants with complete lipoprotein and follow-up data were included in this study (Supplementary file 1: eFigure 1).

### Cox regression analysis

Continuous variables were described using the median and interquartile range, while categorical variables were described using frequencies and percentages. The Kruskal–Wallis and Chi-square tests were employed to compare the continuous variables and categorical variables, respectively, between the groups.

Cox proportional hazard models, with hazard ratio (HR) and 95% confidence interval (CI), were used to assess the relationships between lipoprotein subclasses [per standard deviation (SD) increase] and the risk of GI cancers. Two models were constructed. Model 1 was adjusted based on age and sex, while Model 2 was further adjusted based on ethnicity, BMI, history of chronic diseases, diet score, alcohol and smoking status, lipid-lowering drug treatment, MET, TC, TG, and TDI. The selection of covariates was guided by a directed acyclic graph (DAG), constructed using DAGitty^1^, to minimize bias and overfitting during model adjustment (Supplementary file 1: eFigure 2) (20).

The non-linear relationships between lipoprotein subclasses and GI cancers were analyzed using restricted cubic splines (RCS), adjusted for the maximum covariates in Model 2 (21). The non-linear *p*-values were identified by the log-likelihood ratio. Additionally, stratified analyses were conducted based on several key subgroups, including sex (men/women), age (< or ≥60 y), and BMI (< or ≥30 kg/m^2^). Likelihood ratio tests were used to calculate the *p*-values for between-group interactions.

Several sensitivity analyses were conducted on the relationships between lipoprotein subclasses and the risk of GI cancers. Firstly, participants who developed GI cancer within 2 years of the follow-up period were excluded from this study to avoid potential reverse causal effects. Secondly, the analyses were repeated after excluding participants with any missing covariate values at baseline. In addition, random forest imputation was used to address missing values, and a subset of the data was randomly selected for repeated analysis to explore potential differences among different imputation methods. Finally, considering the potential impact of menopausal status and proton pump inhibitor use on GI cancers, we made further adjustments to validate the robustness of our results.

### Mendelian randomization (MR) analysis

The genome-wide association study (GWAS) data on 17 lipoprotein subclasses and 6 GI cancers were extracted separately for MR analysis. The data all extracted from the IEU GWAS database^2^ (Supplementary file 2: eTable 1). The single-nucleotide polymorphisms (SNPs) that were strongly associated with the exposure were selected as instrumental variables (IVs) for the MR analysis (Supplementary file 2: eTable 2). Various methods were employed to assess the causal relationships between exposure and outcome, including inverse variance weighted (IVW), MR Egger, Wald ratio, weighted median, simple mode, and weighted mode analyses. The specific methods and parameters for MR analysis are detailed in Supplementary file 2.

### Statistical analysis

All statistical analyses were performed using the R software “EmpowerStats” (v4.2.0^3^, X&Y Solutions, Inc., Boston, MA). A two-sided *p*-value of <0.05 was considered statistically significant, with *, **, and *** denoting *p*-value <0.05, <0.01, and <0.001, respectively.

## Results

From a total of 502,357 recorded participants, 249,450 cancer-free participants with complete lipoprotein data were included in this study (Table 1). And 4,787 cases of GI cancers were recorded over the mean follow-up period of 12.92 years, which included 528 EC cases, 338 GC cases, 121 SIC cases, 2,872 CRC cases, 327 LC cases, and 601 PC cases (Table 1).

**Table 1:** Baseline demographic and clinical characteristics in the study.

Characteristic	Total (*n* = 249,450)	No gastrointestinal cancers (*n* = 244,663)	Gastrointestinal cancers (*n* = 4,787)	*P*-value
Age, years	57.0 (50.0–63.0)	57.0 (50.0–63.0)	62.0 (56.0–66.0)	<0.001
Male, *N* (%)	117,528 (47.1%)	114,675 (46.87%)	2,853 (59.6%)	<0.001
White, *N* (%)	236,557 (94.8%)	231,923 (94.79%)	4,634 (96.9%)	<0.001
MET	2666.0 (1066.5–2906.5)	2666.0 (1070.0–2906.5)	2669.0 (990.0–2912.0)	0.17
TDI	−2.18 (−3.67–0.47)	−2.19 (−3.67–0.46)	−2.07 (−3.60–0.80)	<0.001
BMI (kg/m^2^)	26.8 (24.2–29.9)	26.8 (24.2–29.9)	27.5 (24.9–30.7)	<0.001
TC	4.6 (3.99–5.23)	4.6 (3.99–5.23)	4.48 (3.79–5.17)	<0.001
TG	1.20 (0.88–1.64)	1.20 (0.88–1.64)	1.30 (0.94–1.73)	<0.001
Diet score	5 (4–6)	5 (4–6)	5 (4–6)	<0.001
DM	13,086 (5.25%)	12,637 (5.17%)	449 (9.38%)	<0.001
CVD, *N* (%)	19,734 (7.91%)	19,208 (7.85%)	526 (10.99%)	<0.001
Lipid-lowering drugs, *N* (%)	43,752 (17.54%)	42,494 (17.37%)	1,258 (26.28%)	<0.001
Alcohol status, *N* (%)				<0.001
Never	10,845 (4.35%)	10,664 (4.36%)	181 (3.78%)	
Previous	8,792 (3.52%)	8,567 (3.5%)	225 (4.7%)	
Current	229,813 (92.13%)	225,432 (92.14%)	4,381 (91.52%)	
Smoking status, *N* (%)				<0.001
Never	100,279 (40.2%)	98,719 (40.35%)	1,560 (32.59%)	
Previous	123,027 (49.32%)	120,393 (49.21%)	2,634 (55.02%)	
Current	26,144 (10.48%)	25,551 (10.44%)	593 (12.39%)	
HDL-P (μmol/l)	15.12 (13.6–16.8)	15.13 (13.6–16.8)	14.87 (13.29–16.54)	<0.001
VL-HDL-P (μmol/l)	0.21 (0.17–0.27)	0.21 (0.17–0.27)	0.20 (0.16–0.26)	<0.001
L-HDL-P (μmol/l)	1.22 (0.82–1.81)	1.22 (0.82–1.81)	1.10 (0.76–1.66)	<0.001
M-HDL-P (μmol/l)	3.81 (3.24–4.46)	3.81 (3.23–4.46)	3.72 (3.15–4.37)	<0.001
S-HDL-P (μmol/l)	9.72 (8.89–10.60)	9.72 (8.89–10.60)	9.65 (8.77–10.56)	<0.001
IDL-P (μmol/l)	0.30 (0.25–0.36)	0.30 (0.25–0.36)	0.30 (0.24–0.35)	<0.001
LDL-P (μmol/l)	1.18 (1.00–1.38)	1.18 (1.00–1.38)	1.16 (0.97–1.36)	<0.001
L-LDL-P (μmol/l)	0.72 (0.61–0.85)	0.73 (0.61–0.85)	0.71 (0.59–0.84)	<0.001
M-LDL-P (μmol/l)	0.29 (0.24–0.34)	0.29 (0.24–0.34)	0.29 (0.23–0.34)	0.004
S-LDL-P (μmol/l)	0.17 (0.14–0.19)	0.17 (0.14–0.19)	0.17 (0.14–0.19)	0.008
VLDL-P (μmol/l)	0.14 (0.11–0.17)	0.14 (0.11–0.17)	0.14 (0.11–0.17)	0.002
CEL-VLDL-P (nmol/l)	1.25 (0.5–2.43)	1.25 (0.5–2.42)	1.50 (0.66–2.73)	<0.001
VL-VLDL-P (nmol/l)	3.10 (1.75–4.86)	3.09 (1.75–4.85)	3.48 (1.99–5.21)	<0.001
L-VLDL-P (nmol/l)	9.49 (6.07–13.8)	9.49 (6.07–13.8)	10.3 (6.60–14.40)	<0.001
M-VLDL-P (nmol/l)	33.7 (25.9–42.6)	33.7 (25.9–42.6)	33.7 (25.8–42.6)	0.516
S-VLDL-P (nmol/l)	38.3 (30.1–47.8)	38.3 (30.1–47.7)	39.4 (31.2–48.5)	<0.001
VS-VLDL-P (nmol/l)	53.4 (45.1–63.1)	53.4 (45.1–63.1)	53.4 (44.5–63.4)	0.502

MET, metabolic equivalent of task; TDI, townsend deprivation index; BMI, body mass index; TC, total cholesterol; TG, triglycerides; DM, diabetes mellitus; CVD, cardiovascular disease; VL-HDL-P, very large HDL particles; L-HDL-P, large HDL articles; M-HDL-P, medium HDL particles; S-HDL-P, small HDL particles; L-LDL-P, large LDL particles; M-LDL-P, medium LDL particles; S-LDL-P, small LDL particles; CEL-VLDL-P, chylomicrons and extremely large VLDL particles; VL-VLDL-P, very large VLDL particles; L-VLDL-P, large VLDL particles; M-VLDL-P, medium VLDL particles; S-VLDL-P, small VLDL particles; VS-VLDL-P, very small VLDL particles.

### Association between the lipoprotein subclasses and risk of GI cancers

**Figure 1:**
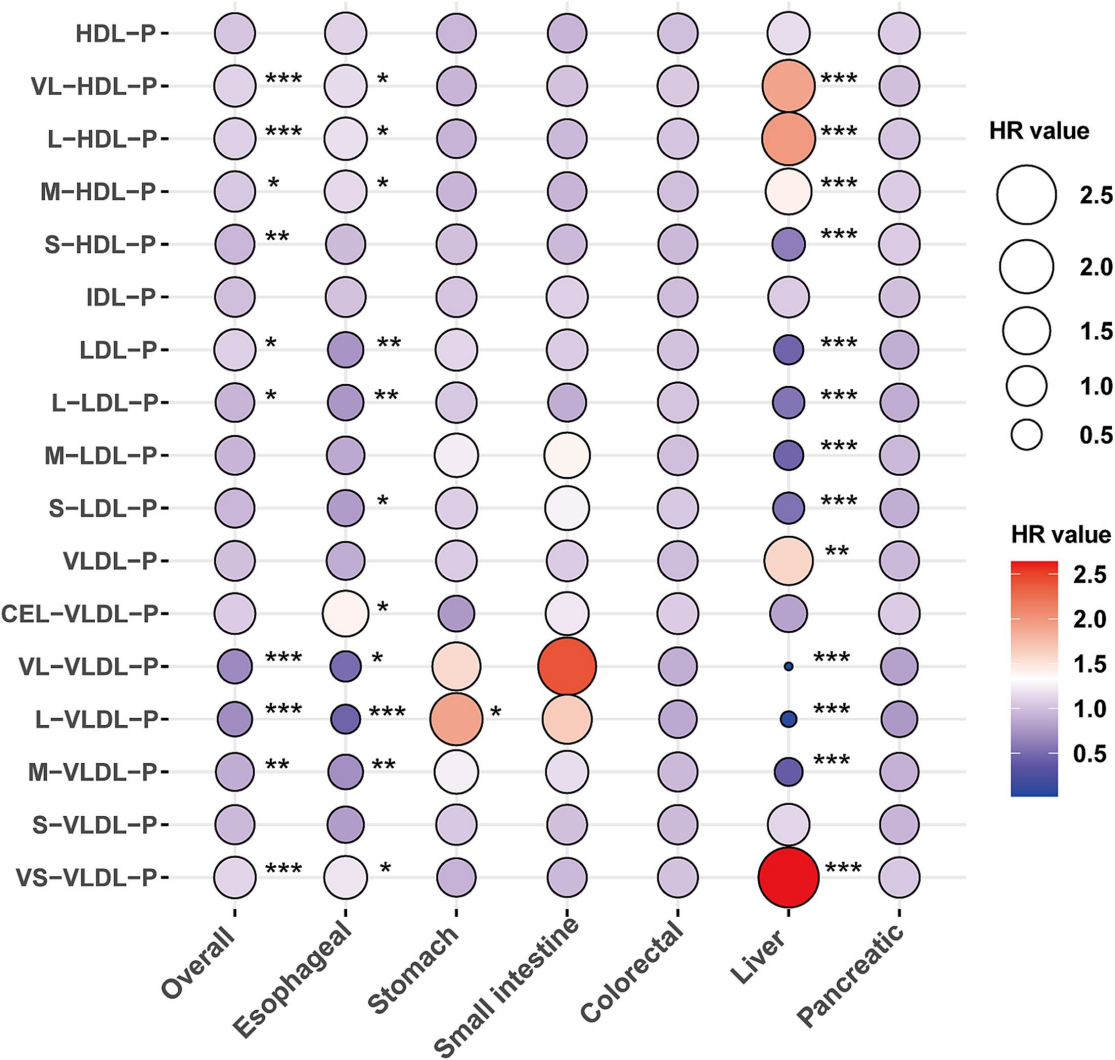
The association between lipoprotein particles and the risk of gastrointestinal cancers. Models were fully adjusted with age, sex, ethnicity, BMI, history of cardiovascular disease (CVD), diabetes mellitus (DM), diet score, alcohol status, smoking status, lipid-lowing drugs, MET, TC, TG, and Townsend deprivation index. *, **, and *** denoting *p* < 0.05, *p* < 0.01, and *p* < 0.001, respectively.

After adjusting for age and sex, 8 and 3 of the 17 lipoprotein subclasses demonstrated significant negative and positive associations, respectively, with the risk of gastrointestinal (GI) cancers in Model 1 (Supplementary file 1: eTable 4). In the adjusted model, each standard deviation (SD) increase in very large high-density lipoprotein particles (VL-HDL-P), large high-density lipoprotein particles (L-HDL-P), and medium high-density lipoprotein particles (M-HDL-P) levels exhibited a positive correlation with the risk of overall GI cancer, esophageal cancer (EC), and liver cancer (LC) (Figure 1 and Supplementary file 1: eTable 4). Conversely, an increase in small high-density lipoprotein particles (S-HDL-P) levels was negatively correlated with the risk of overall GI cancer and LC occurrence.

Additionally, higher levels of low-density lipoprotein particles (LDL-P) were positively associated with the risk of overall GI cancer but inversely associated with the risk of EC and LC. Specifically, large low-density lipoprotein particles (L-LDL-P), medium low-density lipoprotein particles (M-LDL-P), and small low-density lipoprotein particles (S-LDL-P) levels showed a negative correlation with the risk of overall GI cancer, EC, and LC (Figure 1 and Supplementary file 1: eTable 4). Moreover, very large very low-density lipoprotein particles (VL-VLDL-P), large very low-density lipoprotein particles (L-VLDL-P), and medium very low-density lipoprotein particles (M-VLDL-P) demonstrated significant negative correlations, whereas small very low-density lipoprotein particles (VS-VLDL-P) were positively correlated with the risk of overall GI cancer, EC, and LC (Figure 1 and Supplementary file 1: eTable 4).

Among gastric cancer (GC), small intestinal cancer (SIC), colorectal cancer (CRC), and pancreatic cancer (PC), elevated L-VLDL-P levels were significantly associated with an increased risk of GC (HR: 1.91, 95% CI: 1.07–3.4; Figure 1 and Supplementary file 1: eTable 4). Subtype analyses of esophageal and liver cancers indicated that the association between lipoproteins and EC was primarily driven by esophageal squamous cell carcinoma (ESCC). Meanwhile, the relationship between lipoproteins and LC was significantly associated with both liver cancer (LC) and hepatocellular carcinoma (HCC) (Supplementary file 1: eTable 5).

### Dose–response relationship between lipoprotein subclasses and the risk of GI cancer

**Figure 2:**
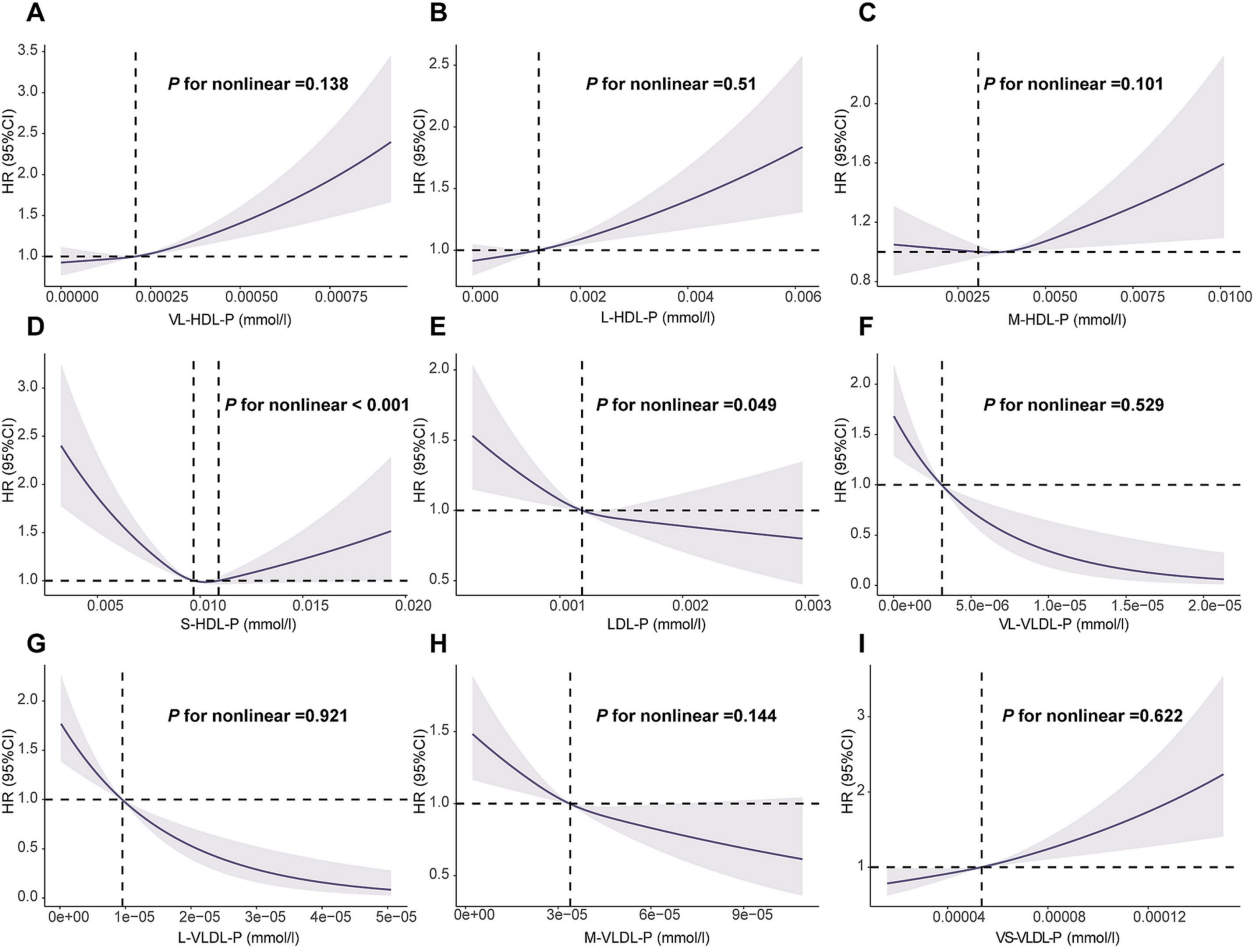
Association of the lipoprotein particles with overall gastrointestinal cancer using RCS with 3 knots. **(A)**: VL-HDL-P, very large HDL particles; **(B)**: L-HDL-P, large HDL articles; **(C)**: M-HDL-P, medium HDL particles; **(D)**: S-HDL-P, small HDL particles; **(E)**: L-LDL-P, large LDL particles; **(F)**: VL-VLDL-P, very large VLDL particles; **(G)**: L-VLDL-P, large VLDL particles; **(H)**: M-VLDL-P, medium VLDL particles; **(I)**: VS-VLDL-P, very small VLDL particles. RCS: restricted cubic spline. Models were fully adjusted with age, sex, ethnicity, BMI, history of cardiovascular disease (CVD), diabetes mellitus (DM), diet score, alcohol status, smoking status, lipid lowing drugs, MET, TC, TG and Townsend deprivation index.

The RCS analysis showed that all the aforementioned lipoprotein particles, except S-HDL-P (*p* < 0.001) and LDL-P (*p* = 0.049), showed a linear dose–response relationship with the risk of overall GI cancer occurrence (*p* > 0.05; Figure 2). Additionally, all lipoprotein particles showed a linear correlation with the risk of EC occurrence (*p* > 0.05; Supplementary file 1: eFigure 3), while S-HDL-P, LDL-P, L-LDL-P, M-LDL-P, and S-LDL-P showed an inverse and non-linear correlation with the risk of LC occurrence (*p* < 0.001; Supplementary file 1: eFigure 4).

### The results of subgroup and sensitivity

Subgroup analysis was conducted based on sex, BMI, and age. The results revealed that the association between lipoprotein particles and the risk of overall GI cancer, EC, and LC occurrence was primarily evident in men. Particularly, HDL-P, VL-HDL-P and VS-VLDL-P levels showed a sex-dependent association with the risk of LC occurrence (*p* < 0.05; Supplementary file 1: eTable 6). In contrast, no association was detected between lipoprotein subclasses and the risk of GI cancers across different BMI groups. However, LDL-P, L-LDL-P, and extremely large-VLDL-particles (CEL-VLDL-P) levels showed an age-dependent association with the risk of EC occurrence (*p* < 0.05; Supplementary file 1: eTable 7), while HDL-P, M-HDL-P, and S-HDL-P showed an age-dependent interaction with the risk of LC occurrence (*p* < 0.05; Supplementary file 1: eTable 8).

Sensitivity analyses showed consistent results after excluding participants who developed GI cancer within 2 years of follow-up and those with missing covariate values at baseline (Supplementary file 1: eTables 9, 10). Additionally, random forest imputation supported the stability of these findings (Supplementary file 1: eTable 11). Finally, our main findings remained robust after additional adjustments for menopausal status and the history of proton pump inhibitor use (Supplementary file 1: eTables 12, 13).

### Causal relationships between lipoprotein subclasses and the risk of GI cancers

**Figure 3:**
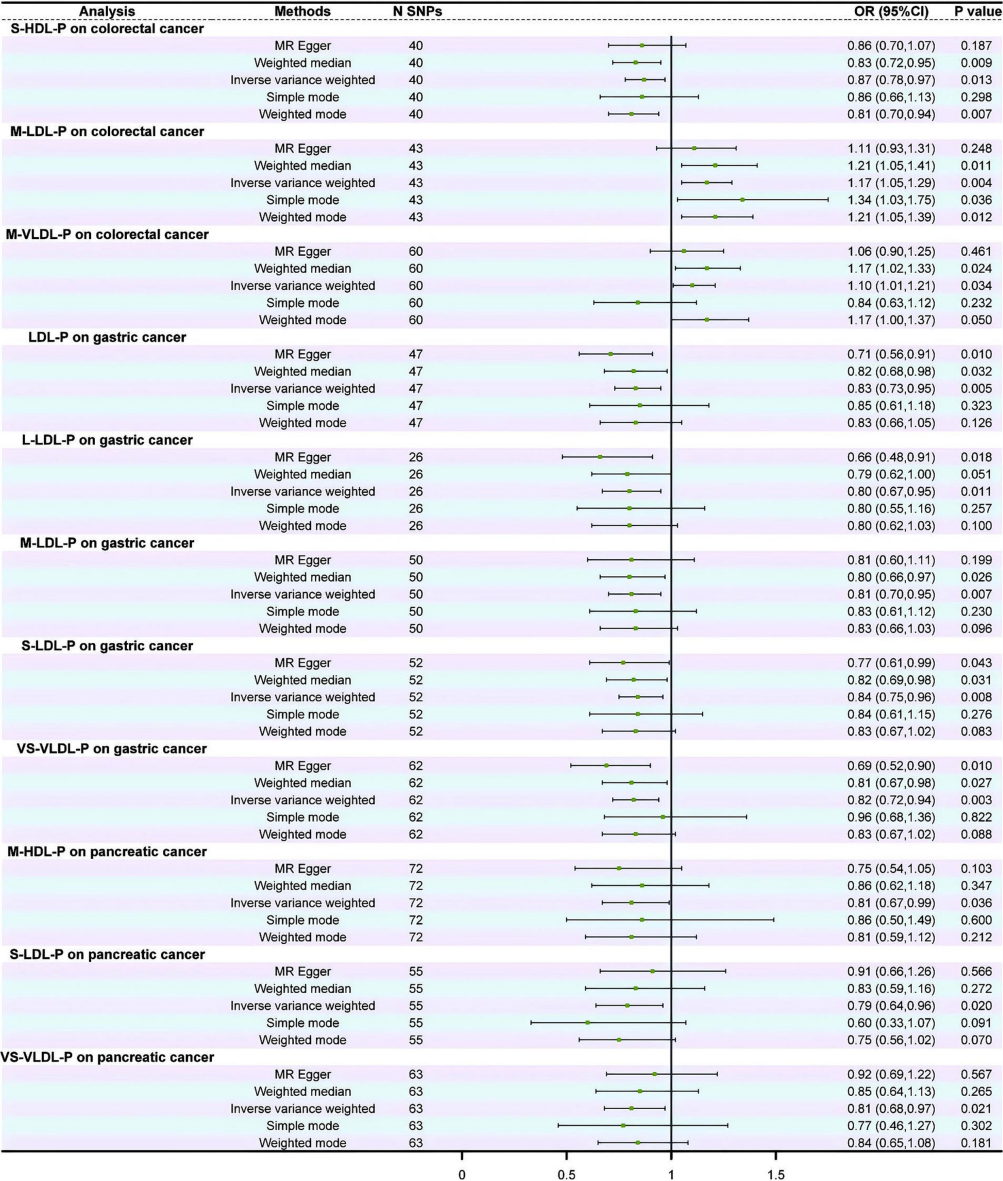
Forest plot of the causal relationship between lipoprotein subclasses and gastrointestinal cancers.

The MR analysis revealed that 11 of the 17 lipoprotein subclasses were causally associated with the risk of CRC, GC, and PC, while no lipoproteins were identified to be causally associated with the risk of EC, LC, and SIC. Moreover, IDL appeared to have no significant correlation with any of the GI cancers (Figure 3 and Supplementary file 2: eTable 3). The IVW results showed that the S-HDL-P subclass served as a protective factor, while the M-LDL-P and M-VLDL-P subclasses served as risk factors for CRC. Additionally, LDL and its subclasses, L-LDL-P, M-LDL-P, and S-LDL-P, were associated with a reduced risk of GC occurrence, while HDL showed no significant correlation with GC occurrence. Moreover, VS-VLDL-P was closely associated with a decreased risk of GC and PC occurrence. Furthermore, M-HDL-P and S-LDL-P served as protective factors in PC (Figure 3 and Supplementary file 2: eTable 3).

The stability of these causal relationships was assessed by Cochran’s IVW *Q* test, which revealed no apparent heterogeneity among the IVs associated with the concentrations of the lipoprotein subclasses (*p* > 0.05; Supplementary file 2: eTable 4). The results of MR-Egger regression intercept analysis and MR pleiotropy residual sum and outlier analysis showed no horizontal pleiotropy (*p* > 0.05; Supplementary file 2: eTable 5) or outliers (global test *p* > 0.05; Supplementary file 2: eTable 6).

## Discussion

In this study, we found that lipoprotein subclasses play significant roles in the development of GI cancers. Specifically, we found that several lipoprotein subclasses were positively associated with the risk of GI occurrence, except for certain HDL subclasses, such as S-HDL-P, which were negatively associated with the risk of GI cancer occurrence. We further elucidated the gender- and age-dependent associations between lipoprotein subclasses and the risk of GI cancers by conducting dose–response and subgroup analyses. The results revealed that M-HDL-P and S-HDL-P exhibited protective effects against CRC and PC, while LDL subclasses and VS-VLDL-P were associated with a decreased risk of GC. However, the MR results did not support the causal association of Cox regression between the lipoprotein subclasses and the risk of GI cancers, at the genetic level.

The findings of this study enhance our understanding of the relationship between lipoprotein subclasses and the risk of gastrointestinal (GI) cancers. Previous research has highlighted the potential roles of lipoproteins in cancer development. For example, Guan et al. (22) and Zhu et al. (23) demonstrated that low-density lipoprotein (LDL) and high-density lipoprotein (HDL) are associated with breast cancer (BC) progression, suggesting that lipoprotein-targeted therapies may represent a potential avenue for the clinical management of BC. Similarly, Stevanovic et al. (24) and Zeljkovic et al. (25) reported that patients with colorectal cancer (CRC) exhibit an increased proportion of smaller lipoprotein particles and reduced diameters of LDL and HDL, indicating a negative correlation with CRC risk, which aligns with the findings of the present study. Furthermore, Carr et al. (26) and Chen et al. (27) identified associations between HDL and LDL levels and the growth, invasion, and prognosis of hepatocellular carcinoma. However, these studies did not investigate the specific functions and effects of distinct lipoprotein subclasses in relation to other GI cancers.

In the present study, Mendelian randomization (MR) analysis was employed to comprehensively assess the causal relationships between various lipoprotein subclasses and the risk of GI cancers at the genetic level. A comparable study conducted by Wu et al. (28) and Zhang and Liu (29), which also utilized MR analysis, found no significant causal association between serum HDL, LDL, and triglyceride (TG) levels and the risk of upper GI cancers, such as esophageal cancer (EC) and gastric cancer (GC). By contrast, our study identified a negative correlation between LDL subclasses and very small very-low-density lipoprotein particles (VS-VLDL-P) with the risk of GC occurrence.

Several factors may account for these discrepancies. First, the present study performed a more granular analysis of lipoprotein subclasses, further stratifying LDL and VS-VLDL-P into specific particle types. This detailed approach may have revealed associations that were not detectable in broader lipid analyses. Second, population differences likely contributed to the divergent findings. While Wu et al. (28) focused primarily on Asian populations, the present study investigated European populations. Variations in genetic backgrounds and environmental factors affecting lipid metabolism across populations may influence the observed associations. Third, differences in MR methodologies and covariate adjustment strategies may have also influenced the results, particularly in instances where study design or confounding factors were not comprehensively addressed.

Although HDL is generally believed to have protective functions in the body, several studies found that the protective functions of HDL vary between different HDL subclasses, with certain subclasses even lacking protective characteristics (30, 31). For instance, a study found that S-HDL-P primarily confers the protective effects of HDL, such as anti-thrombotic function in human platelets, anti-inflammatory function in the bloodstream, and anti-apoptotic function in endothelial cells (32). This is attributed to the significant heterogeneity of HDL particles in circulation, with their different physicochemical properties imparting distinct biological characteristics (33). Rached et al. (34) and Asztalos et al. (35) found that HDL particles undergo dynamic reshaping during the transport of cholesterol and lipids between cells, tissues, and organs. Additionally, a study found that the physicochemical properties and activities of HDL particles can change during systemic inflammation, oxidative stress, and immune dysfunction (31, 36). Cancer cells, especially high-proliferating cancer cells, typically store cholesterol in the form of cholesterol esters; however, the accumulation of cholesterol esters prevents the efflux of cholesterol by S-HDL-P, leading to immature S-HDL-P particles, which may further lead to cancer cell growth (37, 38). Furthermore, the high cholesterol demand by cancer cells leads to high absorption of LDL particles on the cancer cell surface. However, S-LDL-P has lower affinity compared to L-LDL-P, resulting in longer plasma residence time. Moreover, S-LDL-P is prone to oxidation and can be converted to oxidized LDL. Previous studies found that the binding of oxidized LDL to oxidized LDL receptor 1 promotes CRC progression (39, 40). These reports reveal the mechanistic impact of different subclasses of lipoprotein particles on cancer development, providing the scientific basis for further research on the relationship between lipoprotein particles and GI cancers. However, information on the role of VLDL subclasses in GI cancers is limited. In this study, we found that only VS-VLDL-P, among the VLDL subclasses, was significantly associated with the risk of GI cancers, which may be attributed to its larger volume, charge, and lipid content.

However, our study has certain limitations. First, this study is fundamentally observational and provides only the correlation data between the lipoprotein subtypes and the risk of GI cancers and does not explore the molecular mechanisms underlying these associations. Second, the predominant representation of individuals of Caucasian descent in the UKB limits the generalizability of the results. Third, despite multiple adjustments and subgroup analyses, potential confounding factors and assumptions inherent in MR analysis restrict the interpretation and generalizability of the results. Additionally, our study primarily examined baseline levels of 17 lipoprotein subclasses and their associations with GI cancer risk, without addressing temporal changes in high-density lipoprotein cholesterol (HDL-C) levels or their interactions with sex, age, or smoking status. Investigating these dynamic factors was beyond the scope of the present study.

In conclusion, we found a complex association between lipoprotein subclasses and the risk of GI cancers, with different subclasses exerting distinct effects on various types of GI cancers. These findings provide novel insights into further understanding the relationship between lipoproteins and GI cancers, offering potential avenues for GI cancer prevention and treatment.

## Data Availability

The original contributions presented in the study are included in the article/Supplementary material, further inquiries can be directed to the corresponding authors.

## Footnotes

^1^ www.dagitty.net

^2^ www.gwas.mrcieu.ac.uk

^3^ www.R-project.org
