# Supplementary material for: Lipoprotein subclasses and gastrointestinal cancers: novel perspectives and potential associations
Source: Front Nutr. 2025 Mar 3;12:1501263. doi: 10.3389/fnut.2025.1501263 (PMC11912509; doi:10.3389/fnut.2025.1501263)
Supplement: Supplementary file 2 [file Table_1.docx]

**Lipoprotein Subclasses and Gastrointestinal Cancers: Novel Perspectives and Potential Associations**

[eTable 1. ICD-10 codes for Gastrointestinal Cancer 2](#_Toc186573395)

[eTable 2. Histological subtypes of cancer in UK Biobank 3](#_Toc186573396)

[eTable 3. Scoring criteria of diet in UK Biobank according to the Diet score 4](#_Toc186573397)

[eTable 4. Hazard ratios (95% confidence intervals) of the association between lipoprotein particles and the risk of gastrointestinal cancer 5](#_Toc186573398)

[eTable 5. The association between lipoprotein particles and the risk of gastrointestinal cancer in esophageal cancer and liver cancer subtypes. 7](#_Toc186573399)

[eTable 6. Subgroup and interaction analysis between lipoprotein particles and the risk of gastrointestinal cancer across sex 9](#_Toc186573400)

[eTable 7. Subgroup and interaction analysis between lipoprotein particles and the risk of gastrointestinal cancer across BMI 12](#_Toc186573401)

[eTable 8 Subgroup and interaction analysis between lipoprotein particles and the risk of gastrointestinal cancer across age 15](#_Toc186573402)

[eTable 9. Sensitivity analysis between lipoprotein particles and the risk of gastrointestinal cancer with participants exclude gastrointestinal cancer occurred within 2 years 18](#_Toc186573403)

[eTable 10. Sensitivity analysis between lipoprotein particles and the risk of gastrointestinal cancer with participants exclude all missing values at baseline 19](#_Toc186573404)

[eTable 11. Sensitivity analysis between lipoprotein particles and the risk of gastrointestinal cancer with random forest imputation 20](#_Toc186573405)

[eTable 12. Sensitivity analysis between lipoprotein particles and the risk of gastrointestinal cancer after further adjusted menopausal status and history of proton pump inhibitor use. 21](#_Toc186573406)

[eTable 13. The association between lipoprotein particles and the risk of gastrointestinal cancer in esophageal cancer and liver cancer subtypes. 22](#_Toc186573407)

[eFigure 1. Study Flowchart 24](#_Toc186573408)

[eFigure 2. Directed Acyclic Graph for Covariate Selection 25](#_Toc186573409)

[eFigure 3. Association of the lipoprotein particles with esophageal and stomach cancer risk using RCS with 3 knots 26](#_Toc186573410)

[eFigure 4. Association of the lipoprotein particles with liver cancer risk using RCS with 3 knots 27](#_Toc186573411)

| eTable 1. ICD-10 codes for Gastrointestinal Cancer | |
| --- | --- |
| **Cancer Type** | **ICD-10 codes** |
| Overall | C15-C20, C22, C25 |
| Esophageal | C15 |
| Stomach | C16 |
| Small intestine | C17 |
| Colorectal | C18, C19, C20 |
| Liver | C22 |
| Pancreatic | C25 |

| eTable 2. Histological subtypes of cancer in UK Biobank | | |
| --- | --- | --- |
| **Cancer Type** | **Histological subtype** | **UK Biobank data field 40011 values** |
| Esophageal cancer | Adenocarcinoma (EAC) | 8140, 8144, 8210, 8260, 8323, 8480, 8211, 8574, 8481, 8145, 8490 |
|  | Squamous cell carcinoma (ESCC) | 8070, 8071,8072,8076 |
|  | Other types | 8000, 8010, 8020, 8246, 8560, 8012, 8041, 8720, 8990, 8045 |
| Liver cancer | Hepatocellular carcinoma (HCC) | 8170, 8033 |
|  | Cholangiocarcinoma (CAC) | 8140, 8160. 8162, 8180,8481 |
|  | Other types | 8000, 8010, 8480, 9120, 9133, 8890, 8241, 9491, 8240, 8246 |

| eTable 3. Scoring criteria of diet in UK Biobank according to the Diet score | | |
| --- | --- | --- |
| **Diet component** | **Scoring Criteria** | **Score** |
| Fruit & vegetables | <5 serving/day | 1: If scoring condition met  0: If scoring condition not met (Range: 0-9) |
| Total fish intake | < once a week of each one |  |
| Processed meat | > Once a week |  |
| Red meat | ≤Once a week |  |
| Milk type used | Full cream/ another type of milk/ never rarely have milk |  |
| Spread type | Another selection |  |
| Cereal intake | ≤5 bowls |  |
| Salt added to food | Sometimes/usually/always |  |
| Water intake | < 6glasses |  |

| eTable 4. Hazard ratios (95% confidence intervals) of the association between lipoprotein particles and the risk of gastrointestinal cancer | | | | | | | | |
| --- | --- | --- | --- | --- | --- | --- | --- | --- |
| **Type** | **Overall** | | **Esophageal** | | **Stomach** | | **Small intestine** | |
|  | **Model 1** | **Model 2** | **Model 1** | **Model 2** | **Model 1** | **Model 2** | **Model 1** | **Model 2** |
| HDL-P | **0.95 (0.92-0.98)** | 1.02 (0.98-1.06) | 0.98 (0.9-1.08) | 1.1 (0.98-1.24) | **0.84 (0.75-0.95)** | 0.95 (0.82-1.11) | 0.86 (0.71-1.05) | 0.93 (0.73-1.2) |
| VL-HDL-P | 0.97 (0.94-1) | **1.1 (1.06-1.14)** | 1.01 (0.91-1.11) | **1.15 (1.02-1.29)** | **0.82 (0.71-0.94)** | 0.94 (0.8-1.1) | 0.85 (0.68-1.05) | 1.01 (0.79-1.3) |
| L-HDL-P | **0.95 (0.92-0.99)** | **1.09 (1.05-1.13)** | 0.99 (0.9-1.1) | **1.17 (1.04-1.33)** | **0.8 (0.7-0.92)** | 0.93 (0.79-1.1) | **0.78 (0.63-0.98)** | 0.96 (0.74-1.26) |
| M-HDL-P | 0.97 (0.94-1) | **1.04 (1-1.07)** | 1.03 (0.94-1.14) | **1.14 (1.03-1.26)** | **0.84 (0.75-0.96)** | 0.94 (0.82-1.08) | 0.84 (0.69-1.03) | 0.93 (0.74-1.16) |
| S-HDL-P | **0.95 (0.92-0.98)** | **0.95 (0.91-0.99)** | 0.96 (0.88-1.04) | 0.97 (0.86-1.09) | 0.91 (0.82-1.02) | 1 (0.87-1.16) | 0.97 (0.81-1.16) | 0.96 (0.76-1.22) |
| IDL-P | **0.94 (0.91-0.97)** | 0.99 (0.93-1.06) | 0.97 (0.89-1.06) | 1.01 (0.83-1.23) | **0.87 (0.78-0.98)** | 1.02 (0.8-1.3) | 0.97 (0.81-1.16) | 1.09 (0.74-1.62) |
| LDL-P | **0.94 (0.92-0.97)** | **1.09 (0.86-0.99)** | 0.93 (0.86-1.02) | **0.75 (0.6-0.92)** | 0.9 (0.81-1.01) | 1.12 (0.85-1.48) | 1.01 (0.85-1.21) | 1.06 (0.67-1.67) |
| L-LDL-P | **0.94 (0.91-0.96)** | **0.93 (0.87-0.99)** | 0.92 (0.84-1) | **0.76 (0.62-0.92)** | **0.89 (0.79-0.99)** | 1.04 (0.81-1.33) | 0.96 (0.81-1.15) | 0.89 (0.6-1.34) |
| M-LDL-P | **0.96 (0.93-0.99)** | **0.94 (0.88-1)** | 0.96 (0.88-1.05) | 0.87 (0.72-1.05) | 0.94 (0.84-1.05) | 1.24 (0.97-1.59) | 1.09 (0.92-1.3) | 1.39 (0.92-2.08) |
| S-LDL-P | **0.96 (0.94-0.99)** | 0.95 (0.89-1.02) | 0.96 (0.88-1.04) | **0.79 (0.64-0.97)** | 0.92 (0.83-1.03) | 1.08 (0.83-1.42) | 1.08 (0.9-1.28) | 1.27 (0.81-1.98) |
| VLDL-P | 1.02 (0.99-1.05) | 1 (0.92-1.09) | 1.03 (0.94-1.12) | 0.89 (0.69-1.17) | 0.98 (0.88-1.09) | 1.06 (0.76-1.48) | 1.13 (0.95-1.34) | 1.06 (0.62-1.81) |
| CEL-VLDL-P | **1.09 (1.06-1.12)** | 1.06 (0.96-1.17) | **1.13 (1.04-1.22)** | **1.4 (1.04-1.87)** | 1.08 (0.97-1.19) | 0.77 (0.53-1.12) | **1.24 (1.07-1.45)** | 1.21 (0.67-2.19) |
| VL-VLDL-P | **1.07 (1.04-1.1)** | **0.68 (0.55-0.83)** | 1.08 (0.99-1.18) | **0.51 (0.28-0.93)** | 1.08 (0.98-1.2) | 1.57 (0.72-3.44) | **1.25 (1.06-1.47)** | 2.41 (0.67-8.63) |
| L-VLDL-P | **1.05 (1.02-1.08)** | **0.7 (0.61-0.81)** | 1.05 (0.97-1.15) | **0.45 (0.29-0.69)** | 1.08 (0.97-1.2) | **1.91 (1.07-3.4)** | **1.23 (1.05-1.46)** | 1.66 (0.66-4.22) |
| M-VLDL-P | 0.98 (0.95-1.01) | **0.89 (0.82-0.96)** | 0.97 (0.89-1.06) | **0.71 (0.56-0.89)** | 0.97 (0.87-1.08) | 1.25 (0.92-1.68) | 1.11 (0.93-1.32) | 1.16 (0.72-1.87) |
| S-VLDL-P | 1.02 (0.99-1.05) | 0.96 (0.89-1.03) | 1.02 (0.93-1.11) | 0.8 (0.64-1.01) | 1 (0.9-1.12) | 1.04 (0.78-1.39) | 1.15 (0.97-1.36) | 1 (0.63-1.6) |
| VS-VLDL-P | 1.01 (0.98-1.04) | **1.11 (1.05-1.17)** | 1.06 (0.97-1.15) | **1.2 (1.02-1.41)** | 0.9 (0.8-1.01) | 0.92 (0.74-1.13) | 1.03 (0.86-1.23) | 0.96 (0.68-1.35) |

| **Continued eTable 4. Hazard ratios (95% confidence intervals) of the association between lipoprotein particles and the risk of gastrointestinal cancer** | | | | | | |
| --- | --- | --- | --- | --- | --- | --- |
| **Type** | **Colorectal** | | **Liver** | | **Pancreatic** | |
|  | **Model 1** | **Model 2** | **Model 1** | **Model 2** | **Model 1** | **Model 2** |
| HDL-P | 0.97 (0.93-1.01) | 0.99 (0.94-1.04) | **0.78 (0.69-0.88)** | 1.16 (0.99-1.35) | 0.97 (0.89-1.06) | 1.07 (0.96-1.2) |
| VL-HDL-P | 0.97 (0.94-1.02) | 1.05 (1-1.1) | **1.23 (1.11-1.37)** | **1.91 (1.71-2.14)** | **0.9 (0.82-0.98)** | 1 (0.9-1.11) |
| L-HDL-P | 0.96 (0.92-1) | 1.03 (0.98-1.08) | **1.16 (1.03-1.31)** | **1.98 (1.73-2.27)** | **0.91 (0.83-1)** | 1.03 (0.92-1.15) |
| M-HDL-P | 0.97 (0.93-1.01) | 0.99 (0.95-1.04) | 1 (0.88-1.12) | **1.41 (1.23-1.61)** | 0.99 (0.91-1.08) | 1.07 (0.97-1.18) |
| S-HDL-P | 0.99 (0.95-1.02) | 0.96 (0.92-1.01) | **0.6 (0.54-0.67)** | **0.61 (0.53-0.71)** | 1.01 (0.93-1.1) | 1.06 (0.95-1.17) |
| IDL-P | 0.97 (0.94-1.01) | 0.98 (0.9-1.06) | **0.72 (0.64-0.81)** | 1.06 (0.82-1.37) | 0.92 (0.85-1) | 1 (0.84-1.2) |
| LDL-P | 0.99 (0.96-1.03) | 1.01 (0.92-1.11) | **0.64 (0.57-0.72)** | **0.46 (0.35-0.6)** | 0.93 (0.86-1.01) | 0.9 (0.73-1.08) |
| L-LDL-P | 0.99 (0.95-1.02) | 1.02 (0.93-1.1) | **0.64 (0.57-0.72)** | **0.56 (0.43-0.72)** | **0.92 (0.85-1)** | 0.89 (0.74-1.07) |
| M-LDL-P | 0.99 (0.96-1.03) | 0.99 (0.91-1.07) | **0.64 (0.57-0.73)** | **0.46 (0.36-0.59)** | 0.95 (0.88-1.03) | 0.96 (0.8-1.14) |
| S-LDL-P | 1 (0.97-1.04) | 1.04 (0.95-1.13) | **0.69 (0.62-0.78)** | **0.54 (0.42-0.7)** | 0.95 (0.88-1.03) | 0.9 (0.74-1.09) |
| VLDL-P | 1.02 (0.99-1.06) | 0.98 (0.87-1.09) | 0.93 (0.84-1.05) | **1.6 (1.13-2.28)** | 1.02 (0.94-1.1) | 0.96 (0.75-1.22) |
| CEL-VLDL-P | **1.07 (1.03-1.11)** | 1.07 (0.94-1.22) | **1.15 (1.05-1.27)** | 0.83 (0.57-1.21) | **1.11 (1.03-1.19)** | 1.06 (0.8-1.4) |
| VL-VLDL-P | **1.06 (1.02-1.1)** | 0.9 (0.7-1.17) | 1.03 (0.93-1.15) | **0.02 (0.01-0.05)** | **1.09 (1.01-1.18)** | 0.82 (0.47-1.43) |
| L-VLDL-P | **1.05 (1.01-1.09)** | 0.87 (0.72-1.05) | 0.96 (0.86-1.08) | **0.08 (0.05-0.12)** | 1.07 (0.99-1.16) | 0.77 (0.51-1.15) |
| M-VLDL-P | 1.01 (0.97-1.05) | 0.96 (0.87-1.06) | **0.72 (0.64-0.82)** | **0.41 (0.31-0.55)** | 0.98 (0.91-1.06) | 0.91 (0.73-1.12) |
| S-VLDL-P | 1.03 (0.99-1.07) | 0.97 (0.88-1.07) | 0.95 (0.85-1.06) | 1.12 (0.83-1.51) | 1.03 (0.95-1.12) | 0.94 (0.76-1.17) |
| VS-VLDL-P | 1 (0.97-1.04) | 1.01 (0.94-1.09) | 1.1 (0.98-1.22) | **2.64 (2.25-3.1)** | 0.98 (0.91-1.07) | 1.04 (0.89-1.21) |

Model 1 was adjusted with age, sex. Model 2 was further adjusted with ethnicity, BMI, history of cardiovascular disease (CVD), diabetes mellitus (DM), diet score, alcohol status, smoking status, lipid-lowing drugs, MET, TC, TG and Townsend deprivation index. VL-HDL-P: very large HDL particles; L-HDL-P: large HDL articles; M-HDL-P: medium HDL particles; S-HDL-P: small HDL particles; L-LDL-P: large LDL particles; M-LDL-P: medium LDL particles; S-LDL-P: small LDL particles; CEL-VLDL-P: chylomicrons and extremely large VLDL particles; VL-VLDL-P: very large VLDL particles; L-VLDL-P: large VLDL particles; M-VLDL-P: medium VLDL particles; S-VLDL-P: small VLDL particles; VS-VLDL-P: very small VLDL particles.

| eTable 5. The association between lipoprotein particles and the risk of gastrointestinal cancer in esophageal cancer and liver cancer subtypes. |
| --- |

| **Type** | **Esophageal cancer** | | **Liver cancer** | |
| --- | --- | --- | --- | --- |
|  | **EAC** | **ESCC** | **HCC** | **CAC** |
| HDL-P | 0.99 (0.85-1.15) | **1.3 (1.05-1.6)** | 1.02 (0.81-1.28) | **1.39 (1.11-1.73)** |
| VL-HDL-P | 1.09 (0.94-1.28) | 1.18 (0.98-1.42) | **2.61 (2.29-2.97)** | 1.19 (0.96-1.46) |
| L-HDL-P | 1.09 (0.92-1.28) | **1.27 (1.04-1.55)** | **2.8 (2.35-3.34)** | **1.32 (1.06-1.64)** |
| M-HDL-P | 1.05 (0.92-1.19) | **1.31 (1.08-1.58)** | **1.49 (1.23-1.81)** | **1.37 (1.13-1.66)** |
| S-HDL-P | 0.91 (0.79-1.05) | 1.08 (0.88-1.34) | **0.39 (0.32-0.47)** | 1.16 (0.93-1.45) |
| IDL-P | 1.16 (0.91-1.47) | 0.83 (0.58-1.18) | **1.57 (1.08-2.28)** | **0.66 (0.45-0.97)** |
| LDL-P | 0.91 (0.7-1.19) | **0.55 (0.38-0.81)** | **0.38 (0.26-0.57)** | **0.53 (0.36-0.79)** |
| L-LDL-P | 0.9 (0.7-1.15) | **0.58 (0.41-0.82)** | **0.54 (0.37-0.79)** | **0.57 (0.39-0.82)** |
| M-LDL-P | 0.99 (0.78-1.26) | **0.69 (0.49-0.99)** | **0.29 (0.21-0.41)** | **0.66 (0.46-0.95)** |
| S-LDL-P | 0.94 (0.73-1.22) | **0.58 (0.4-0.84)** | **0.48 (0.33-0.69)** | **0.57 (0.39-0.84)** |
| VLDL-P | 1.29 (0.93-1.79) | **0.49 (0.3-0.79)** | **4.22 (2.54-7)** | **0.57 (0.34-0.94)** |
| CEL-VLDL-P | 1.04 (0.73-1.47) | **4.2 (2.32-7.63)** | 0.79 (0.46-1.33) | 0.81 (0.46-1.42) |
| VL-VLDL-P | 0.5 (0.24-1.05) | 0.88 (0.28-2.75) | **0 (0-0.01)** | **0.18 (0.06-0.52)** |
| L-VLDL-P | **0.59 (0.35-0.99)** | 0.31 (0.14-0.71) | **0.02 (0.01-0.04)** | **0.3 (0.14-0.67)** |
| M-VLDL-P | 0.9 (0.68-1.2) | **0.5 (0.33-0.76)** | **0.31 (0.2-0.47)** | **0.5 (0.33-0.76)** |
| S-VLDL-P | 1.1 (0.83-1.45) | **0.41 (0.26-0.64)** | **1.8 (1.16-2.79)** | 0.67 (0.43-1.04) |
| VS-VLDL-P | **1.42 (1.17-1.73)** | 0.84 (0.61-1.16) | **4.19 (3.58-4.92)** | 0.9 (0.65-1.25) |

Models were fully adjusted with age, sex, ethnicity, BMI, history of cardiovascular disease (CVD), diabetes mellitus (DM), diet score, alcohol status, smoking status, lipid-lowing drugs, MET, TC, TG and Townsend deprivation index. VL-HDL-P: very large HDL particles; L-HDL-P: large HDL articles; M-HDL-P: medium HDL particles; S-HDL-P: small HDL particles; L-LDL-P: large LDL particles; M-LDL-P: medium LDL particles; S-LDL-P: small LDL particles; CEL-VLDL-P: chylomicrons and extremely large VLDL particles; VL-VLDL-P: very large VLDL particles; L-VLDL-P: large VLDL particles; M-VLDL-P: medium VLDL particles; S-VLDL-P: small VLDL particles; VS-VLDL-P: very small VLDL particles.

| eTable 6. Subgroup and interaction analysis between lipoprotein particles and the risk of gastrointestinal cancer across sex | | | | | | | | | |
| --- | --- | --- | --- | --- | --- | --- | --- | --- | --- |
| **Type** | **Overall** | |  | **Esophageal** | |  | **Stomach** | |  |
|  | **Male** | **Female** | **P for interaction** | **Male** | **Female** | **P for interaction** | **Male** | **Female** | **P for interaction** |
| HDL-P | 1.03 (0.98-1.09) | 0.99 (0.94-1.06) | 0.285 | 1.11 (0.96-1.29) | 1.04 (0.85-1.29) | 0.203 | 0.93 (0.77-1.13) | 1.02 (0.78-1.32) | 0.501 |
| VL-HDL-P | **1.14 (1.08-1.21)** | 1.04 (0.99-1.1) | 0.818 | **1.19 (1.02-1.39)** | 1 (0.83-1.2) | 0.325 | 0.86 (0.69-1.08) | 1.08 (0.86-1.37) | 0.604 |
| L-HDL-P | **1.14 (1.07-1.21)** | 1.03 (0.97-1.09) | 0.891 | **1.23 (1.04-1.44)** | 1.01 (0.83-1.23) | 0.348 | 0.85 (0.68-1.06) | 1.1 (0.86-1.41) | 0.64 |
| M-HDL-P | **1.07 (1.02-1.12)** | 1 (0.94-1.05) | 0.938 | **1.16 (1.02-1.32)** | 1.06 (0.88-1.27) | 0.481 | 0.92 (0.77-1.09) | 1.01 (0.8-1.27) | 0.485 |
| S-HDL-P | **0.94 (0.89-0.99)** | 0.97 (0.92-1.03) | 0.165 | 0.97 (0.84-1.12) | 1.03 (0.84-1.25) | 0.39 | 1.02 (0.85-1.22) | 0.96 (0.75-1.24) | 0.554 |
| IDL-P | 1.04 (0.96-1.14) | 0.95 (0.86-1.04) | 0.537 | 0.99 (0.78-1.26) | 1.09 (0.79-1.5) | 0.064 | 1.14 (0.84-1.54) | 0.78 (0.51-1.19) | 0.516 |
| LDL-P | **0.86 (0.78-0.95)** | 1.02 (0.91-1.13) | 0.025 | **0.72 (0.55-0.94)** | 0.9 (0.62-1.31) | 0.09 | 1.13 (0.79-1.6) | 1.04 (0.65-1.66) | 0.58 |
| L-LDL-P | **0.88 (0.8-0.96)** | 1.01 (0.92-1.11) | 0.017 | **0.75 (0.59-0.95)** | 0.86 (0.61-1.21) | 0.081 | 1 (0.73-1.36) | 1.04 (0.68-1.6) | 0.553 |
| M-LDL-P | **0.9 (0.83-0.98)** | 1.02 (0.92-1.12) | 0.067 | 0.83 (0.66-1.06) | 1.05 (0.75-1.48) | 0.153 | 1.34 (0.99-1.83) | 1.04 (0.68-1.57) | 0.68 |
| S-LDL-P | **0.9 (0.82-0.99)** | 1.03 (0.93-1.14) | 0.068 | **0.73 (0.57-0.94)** | 0.98 (0.68-1.4) | 0.135 | 1.14 (0.81-1.6) | 0.95 (0.61-1.48) | 0.513 |
| VLDL-P | 1.04 (0.92-1.17) | 0.99 (0.87-1.13) | 0.864 | 0.89 (0.64-1.24) | 1.1 (0.69-1.75) | 0.895 | 1.07 (0.79-1.63) | 0.92 (0.52-1.62) | 0.132 |
| CEL-VLDL-P | 1.1 (0.97-1.24) | 0.99 (0.84-1.17) | 0.22 | 1.32 (0.94-1.85) | 1.47 (0.8-2.71) | 0.075 | 1.03 (0.66-1.62) | 0.41 (0.21-0.82) | 0.154 |
| VL-VLDL-P | **0.59 (0.45-0.77)** | 0.85 (0.63-1.15) | 0.803 | **0.44 (0.21-0.91)** | 0.99 (0.35-2.81) | 0.164 | **3.05 (1.13-8.28)** | 0.45 (0.13-1.56) | 0.07 |
| L-VLDL-P | **0.62 (0.51-0.75)** | 0.89 (0.71-1.12) | 0.694 | **0.44 (0.26-0.72)** | 0.69 (0.3-1.55) | 0.355 | **2.46 (1.19-5.08)** | 1.05 (0.4-2.78) | 0.081 |
| M-VLDL-P | **0.85 (0.77-0.95)** | 0.96 (0.86-1.08) | 0.124 | **0.69 (0.52-0.92)** | 0.88 (0.58-1.33) | 0.39 | 1.33 (0.92-1.93) | 1.01 (0.61-1.66) | 0.343 |
| S-VLDL-P | 0.93 (0.84-1.03) | 1.02 (0.91-1.15) | 0.504 | 0.8 (0.6-1.05) | 0.99 (0.64-1.53) | 0.969 | 0.99 (0.69-1.41) | 1.04 (0.63-1.72) | 0.056 |
| VS-VLDL-P | **1.2 (1.12-1.3)** | 1 (0.92-1.09) | 0.26 | 1.22 (0.99-1.49) | 1.21 (0.91-1.62) | 0.58 | 0.89 (0.68-1.17) | 0.9 (0.63-1.3) | 0.31 |

| **Continued eTable 6. Subgroup and interaction analysis between lipoprotein particles and the risk of gastrointestinal cancer across sex** | | | | | | |
| --- | --- | --- | --- | --- | --- | --- |
| **Type** | **Small intestine** | |  | **Colorectal** | |  |
|  | **Male** | **Female** | **P for interaction** | **Male** | **Female** | **P for interaction** |
| HDL-P | 0.9 (0.63-1.3) | 0.98 (0.69-1.4) | 0.636 | 1.01 (0.94-1.08) | 0.95 (0.88-1.03) | 0.824 |
| VL-HDL-P | 1.18 (0.81-1.73) | 0.9 (0.64-1.27) | 0.668 | 1.03 (0.95-1.11) | 1.04 (0.97-1.11) | 0.194 |
| L-HDL-P | 1.08 (0.72-1.63) | 0.89 (0.62-1.27) | 0.826 | 1.03 (0.95-1.11) | 1.01 (0.94-1.08) | 0.279 |
| M-HDL-P | 0.96 (0.69-1.33) | 0.91 (0.67-1.26) | 0.949 | 1.02 (0.95-1.08) | 0.96 (0.9-1.02) | 0.936 |
| S-HDL-P | 0.84 (0.59-1.18) | 1.12 (0.8-1.57) | 0.345 | 0.99 (0.93-1.06) | 0.94 (0.88-1.01) | 0.717 |
| IDL-P | 1.48 (0.84-2.63) | 0.81 (0.46-1.42) | 0.644 | 0.99 (0.89-1.11) | 0.98 (0.87-1.1) | 0.602 |
| LDL-P | 0.87 (0.45-1.66) | 1.24 (0.65-2.34) | 0.534 | 0.97 (0.85-1.11) | 1.09 (0.96-1.25) | 0.304 |
| L-LDL-P | 0.7 (0.39-1.24) | 1.09 (0.61-1.92) | 0.422 | 0.98 (0.87-1.1) | 1.09 (0.96-1.23) | 0.222 |
| M-LDL-P | 1.43 (0.8-2.57) | 1.34 (0.76-2.37) | 0.81 | 0.98 (0.87-1.1) | 1.03 (0.91-1.16) | 0.537 |
| S-LDL-P | 1.12 (0.59-2.13) | 1.39 (0.75-2.57) | 0.602 | 0.99 (0.88-1.13) | 1.1 (0.97-1.25) | 0.452 |
| VLDL-P | 1.09 (0.5-2.42) | 0.97 (0.45-2.08) | 0.724 | 0.97 (0.83-1.14) | 1.02 (0.87-1.21) | 0.935 |
| CEL-VLDL-P | 1.95 (0.89-4.27) | 0.67 (0.27-1.68) | 0.965 | 1.09 (0.92-1.28) | 1.02 (0.83-1.27) | 0.246 |
| VL-VLDL-P | 2.81 (0.44-18.17) | 2.1 (0.36-12.3) | 0.803 | 0.92 (0.64-1.31) | 0.97 (0.67-1.41) | 0.366 |
| L-VLDL-P | 1.01 (0.28-3.61) | 2.81 (0.7-11.26) | 0.624 | 0.84 (0.65-1.09) | 0.98 (0.73-1.31) | 0.576 |
| M-VLDL-P | 1 (0.5-2) | 1.29 (0.65-2.55) | 0.573 | 0.95 (0.83-1.09) | 1.02 (0.88-1.18) | 0.722 |
| S-VLDL-P | 0.79 (0.41-1.53) | 1.24 (0.63-2.44) | 0.446 | 0.94 (0.83-1.08) | 1.04 (0.9-1.21) | 0.987 |
| VS-VLDL-P | 1.2 (0.74-1.95) | 0.74 (0.45-1.21) | 0.758 | 1.03 (0.93-1.13) | 1.01 (0.91-1.12) | 0.926 |

| **Continued eTable 6. Subgroup and interaction analysis between lipoprotein particles and the risk of gastrointestinal cancer across sex** | | | | | | |
| --- | --- | --- | --- | --- | --- | --- |
| **Type** | **Liver** | |  | **Pancreatic** | |  |
|  | **Male** | **Female** | **P for interaction** | **Male** | **Female** | **P for interaction** |
| HDL-P | 1.09 (0.89-1.32) | **1.32 (1.02-1.71)** | 0.006 | 1.11 (0.94-1.3) | 1.05 (0.9-1.22) | 0.059 |
| VL-HDL-P | **2.36 (2.09-2.67)** | 1.22 (0.98-1.53) | 0.002 | 1 (0.83-1.2) | 1.03 (0.89-1.18) | 0.467 |
| L-HDL-P | **2.47 (2.11-2.9)** | **1.32 (1.04-1.68)** | 0.024 | 1.07 (0.89-1.28) | 1.03 (0.89-1.2) | 0.227 |
| M-HDL-P | **1.45 (1.23-1.71)** | **1.32 (1.05-1.66)** | 0.387 | 1.11 (0.96-1.28) | 1.03 (0.9-1.19) | 0.083 |
| S-HDL-P | **0.48 (0.4-0.58)** | 1.07 (0.83-1.39) | <0.001 | 1.06 (0.91-1.24) | 1.03 (0.89-1.2) | 0.101 |
| IDL-P | 1.38 (0.99-1.9) | 0.68 (0.45-1.05) | 0.11 | 1.07 (0.82-1.39) | 0.96 (0.75-1.22) | 0.031 |
| LDL-P | **0.42 (0.3-0.59)** | **0.56 (0.35-0.89)** | 0.003 | 0.79 (0.59-1.05) | 0.96 (0.73-1.26) | 0.17 |
| L-LDL-P | **0.55 (0.4-0.76)** | **0.57 (0.37-0.88)** | 0.006 | 0.81 (0.62-1.05) | 0.95 (0.74-1.23) | 0.169 |
| M-LDL-P | **0.37 (0.27-0.49)** | 0.73 (0.48-1.12) | <0.001 | 0.87 (0.67-1.13) | 1.02 (0.79-1.3) | 0.199 |
| S-LDL-P | **0.51 (0.37-0.71)** | **0.61 (0.39-0.94)** | 0.01 | 0.85 (0.64-1.12) | 0.93 (0.71-1.2) | 0.169 |
| VLDL-P | **2.72 (1.75-4.24)** | 0.68 (0.38-1.23) | 0.905 | 0.91 (0.63-1.3) | 0.98 (0.7-1.38) | 0.384 |
| CEL-VLDL-P | 0.78 (0.5-1.21) | 0.92 (0.44-1.94) | 0.554 | 1.06 (0.72-1.54) | 1.07 (0.71-1.63) | 0.716 |
| VL-VLDL-P | **0.01 (0-0.02)** | **0.21 (0.06-0.76)** | 0.399 | 0.72 (0.31-1.62) | 0.84 (0.39-1.81) | 0.931 |
| L-VLDL-P | **0.04 (0.02-0.08)** | **0.32 (0.12-0.86)** | 0.12 | 0.68 (0.38-1.22) | 0.79 (0.44-1.43) | 0.978 |
| M-VLDL-P | **0.34 (0.24-0.5)** | **0.58 (0.35-0.97)** | 0.004 | 0.85 (0.62-1.16) | 0.93 (0.69-1.26) | 0.28 |
| S-VLDL-P | 1.36 (0.94-1.97) | 0.81 (0.47-1.39) | 0.817 | 0.84 (0.62-1.14) | 1.02 (0.75-1.39) | 0.706 |
| VS-VLDL-P | **3.54 (3.03-4.14)** | 0.94 (0.64-1.36) | 0.013 | 1.07 (0.86-1.34) | 1.02 (0.82-1.26) | 0.113 |

Models were fully adjusted with age, sex, ethnicity, BMI, history of cardiovascular disease (CVD), diabetes mellitus (DM), diet score, alcohol status, smoking status, lipid-lowing drugs, MET, TC, TG and Townsend deprivation index. VL-HDL-P: very large HDL particles; L-HDL-P: large HDL articles; M-HDL-P: medium HDL particles; S-HDL-P: small HDL particles; L-LDL-P: large LDL particles; M-LDL-P: medium LDL particles; S-LDL-P: small LDL particles; CEL-VLDL-P: chylomicrons and extremely large VLDL particles; VL-VLDL-P: very large VLDL particles; L-VLDL-P: large VLDL particles; M-VLDL-P: medium VLDL particles; S-VLDL-P: small VLDL particles; VS-VLDL-P: very small VLDL particles.

| eTable 7. Subgroup and interaction analysis between lipoprotein particles and the risk of gastrointestinal cancer across BMI | | | | | | | | | |
| --- | --- | --- | --- | --- | --- | --- | --- | --- | --- |
| **Type** | **Overall** | |  | **Esophageal** | |  | **Stomach** | |  |
|  | **≥30 kg/m^2^** | **<30 kg/m^2^** | **P for interaction** | **≥30 kg/m^2^** | **<30 kg/m^2^** | **P for interaction** | **≥30 kg/m^2^** | **<30 kg/m^2^** | **P for interaction** |
| HDL-P | 1.04 (0.96-1.12) | 1 (0.96-1.05) | 0.128 | 1.07 (0.86-1.33) | 1.11 (0.97-1.28) | 0.379 | 0.92 (0.68-1.24) | 0.94 (0.79-1.13) | 0.258 |
| VL-HDL-P | **1.12 (1.02-1.23)** | **1.08 (1.03-1.12)** | 0.292 | 1.28 (1-1.64) | 1.12 (0.98-1.28) | 0.744 | 1.06 (0.73-1.54) | 0.87 (0.72-1.05) | 0.985 |
| L-HDL-P | **1.12 (1.02-1.23)** | **1.07 (1.02-1.12)** | 0.145 | 1.29 (1-1.67) | 1.15 (1-1.33) | 0.738 | 1.03 (0.69-1.51) | 0.87 (0.71-1.05) | 0.735 |
| M-HDL-P | 1.05 (0.98-1.13) | 1.02 (0.98-1.07) | 0.187 | 1.13 (0.93-1.38) | **1.14 (1.01-1.29)** | 0.439 | 0.91 (0.69-1.21) | 0.93 (0.79-1.09) | 0.469 |
| S-HDL-P | 0.98 (0.91-1.05) | **0.95 (0.9-0.99)** | 0.572 | 0.92 (0.74-1.14) | 1 (0.87-1.15) | 0.366 | 0.92 (0.69-1.22) | 1.04 (0.88-1.24) | 0.137 |
| IDL-P | 0.95 (0.84-1.07) | 1.02 (0.95-1.1) | 0.045 | 1.01 (0.71-1.42) | 1 (0.79-1.26) | 0.87 | 1.14 (0.71-1.85) | 1.02 (0.76-1.35) | 0.2 |
| LDL-P | 0.9 (0.78-1.04) | 0.94 (0.87-1.03) | 0.246 | 0.77 (0.51-1.16) | **0.74 (0.58-0.95)** | 0.691 | 1.04 (0.59-1.84) | 1.21 (0.87-1.67) | 0.208 |
| L-LDL-P | 0.91 (0.8-1.03) | 0.95 (0.88-1.03) | 0.161 | 0.79 (0.55-1.15) | **0.74 (0.59-0.94)** | 0.771 | 0.96 (0.58-1.58) | 1.1 (0.82-1.47) | 0.175 |
| M-LDL-P | 0.95 (0.84-1.08) | 0.96 (0.89-1.03) | 0.513 | 0.88 (0.61-1.27) | 0.87 (0.69-1.09) | 0.666 | 1.24 (0.76-2.03) | 1.32 (0.98-1.76) | 0.284 |
| S-LDL-P | 0.93 (0.81-1.06) | 0.97 (0.9-1.05) | 0.491 | 0.79 (0.53-1.17) | 0.79 (0.62-1.01) | 0.537 | 0.99 (0.58-1.69) | 1.16 (0.85-1.59) | 0.327 |
| VLDL-P | 0.97 (0.82-1.15) | 1.04 (0.94-1.15) | 0.974 | 0.94 (0.57-1.55) | 0.87 (0.64-1.2) | 0.248 | 0.93 (0.48-1.8) | 1.17 (0.79-1.74) | 0.919 |
| CEL-VLDL-P | 1.13 (0.96-1.33) | 1.01 (0.89-1.15) | 0.025 | **1.75 (1.07-2.87)** | 1.25 (0.87-1.81) | 0.284 | 0.85 (0.46-1.57) | 0.69 (0.43-1.11) | 0.116 |
| VL-VLDL-P | **0.65 (0.45-0.96)** | **0.72 (0.57-0.92)** | 0.137 | 0.45 (0.15-1.37) | 0.55 (0.27-1.13) | 0.143 | 1.9 (0.41-8.81) | 1.69 (0.67-4.27) | 0.23 |
| L-VLDL-P | **0.62 (0.47-0.81)** | **0.78 (0.65-0.93)** | 0.436 | 0.33 (0.15-0.7) | **0.51 (0.3-0.86)** | 0.061 | 2.16 (0.73-6.34) | **2.14 (1.05-4.35)** | 0.22 |
| M-VLDL-P | **0.83 (0.71-0.96)** | 0.93 (0.85-1.02) | 0.466 | 0.67 (0.44-1.04) | **0.72 (0.55-0.94)** | 0.257 | 1.29 (0.71-2.34) | 1.31 (0.92-1.86) | 0.76 |
| S-VLDL-P | 0.93 (0.8-1.08) | 0.99 (0.91-1.09) | 0.802 | 0.79 (0.52-1.22) | 0.8 (0.61-1.05) | 0.162 | 0.88 (0.51-1.54) | 1.16 (0.82-1.64) | 0.968 |
| VS-VLDL-P | **1.11 (1-1.23)** | **1.11 (1.04-1.19)** | 0.346 | 1.28 (0.96-1.71) | 1.15 (0.94-1.4) | 0.494 | 0.84 (0.56-1.26) | 0.96 (0.75-1.24) | 0.429 |

| **Continued eTable 7. Subgroup and interaction analysis between lipoprotein particles and the risk of gastrointestinal cancer across BMI** | | | | | | | | | | |
| --- | --- | --- | --- | --- | --- | --- | --- | --- | --- | --- |
| **Type** | **Small intestine** | | | |  | **Colorectal** | | | |  |
|  | **≥30 kg/m^2^** | | **<30 kg/m^2^** | | **P for interaction** | **≥30 kg/m^2^** | | **<30 kg/m^2^** | | **P for interaction** |
| HDL-P | 1.21 (0.78-1.89) | | 0.82 (0.61-1.12) | | 0.298 | 0.99 (0.89-1.09) | | 0.98 (0.92-1.04) | | 0.121 |
| VL-HDL-P | **0.47 (0.23-0.94)** | | 1.13 (0.86-1.48) | | 0.054 | 1.05 (0.93-1.18) | | 1.03 (0.98-1.09) | | 0.227 |
| L-HDL-P | 0.66 (0.34-1.29) | | 1.01 (0.74-1.36) | | 0.252 | 1 (0.88-1.14) | | 1.02 (0.96-1.08) | | 0.038 |
| M-HDL-P | 1.13 (0.75-1.71) | | 0.84 (0.64-1.1) | | 0.492 | 0.98 (0.89-1.08) | | 0.99 (0.94-1.04) | | 1 |
| S-HDL-P | 1.43 (0.94-2.18) | | 0.82 (0.61-1.1) | | 0.051 | 0.99 (0.9-1.09) | | 0.96 (0.91-1.02) | | 0.579 |
| IDL-P | 1.19 (0.59-2.36) | | 1.08 (0.67-1.76) | | 0.26 | 0.91 (0.78-1.07) | | 1.01 (0.92-1.12) | | 0.086 |
| LDL-P | 0.7 (0.3-1.61) | | 1.29 (0.75-2.21) | | 0.676 | 1.06 (0.88-1.28) | | 1.02 (0.91-1.13) | | 0.594 |
| L-LDL-P | 0.58 (0.28-1.22) | | 1.08 (0.67-1.76) | | 0.777 | 1.05 (0.89-1.25) | | 1.02 (0.93-1.12) | | 0.464 |
| M-LDL-P | 1.36 (0.63-2.92) | | 1.46 (0.9-2.36) | | 0.445 | 1.02 (0.86-1.2) | | 0.99 (0.9-1.09) | | 0.78 |
| S-LDL-P | 0.84 (0.37-1.89) | | 1.55 (0.91-2.64) | | 0.761 | 1.09 (0.91-1.31) | | 1.03 (0.93-1.14) | | 0.993 |
| VLDL-P | 0.97 (0.36-2.65) | | 1.09 (0.58-2.07) | | 0.865 | 0.9 (0.72-1.13) | | 1.03 (0.9-1.17) | | 0.84 |
| CEL-VLDL-P | 0.98 (0.36-2.69) | | 1.4 (0.68-2.85) | | 0.555 | 1.07 (0.86-1.34) | | 1.05 (0.89-1.23) | | 0.038 |
| VL-VLDL-P | 2.12 (0.2-22.82) | | 3.21 (0.69-14.91) | | 0.86 | 0.96 (0.58-1.61) | | 0.92 (0.68-1.25) | | 0.058 |
| L-VLDL-P | 1.26 (0.23-6.82) | | 2.1 (0.67-6.57) | | 0.89 | 0.84 (0.58-1.2) | | 0.93 (0.74-1.17) | | 0.134 |
| M-VLDL-P | 1.04 (0.41-2.61) | | 1.24 (0.7-2.2) | | 0.692 | 0.89 (0.73-1.08) | | 1.01 (0.9-1.14) | | 0.811 |
| S-VLDL-P | 0.9 (0.38-2.15) | | 1.04 (0.6-1.8) | | 0.962 | 0.92 (0.76-1.12) | | 1.01 (0.9-1.13) | | 0.616 |
| VS-VLDL-P | 0.97 (0.54-1.76) | | 0.94 (0.62-1.42) | | 0.652 | 0.98 (0.85-1.12) | | 1.03 (0.95-1.12) | | 0.383 |
| **Continued eTable 7. Subgroup and interaction analysis between lipoprotein particles and the risk of gastrointestinal cancer across BMI** | | | | | | | | | | |
| **Type** | | **Liver** | |  | | **Pancreatic** | | |  | |
|  |  | **≥30 kg/m^2^** | **<30 kg/m^2^** | **P for interaction** | | **≥30 kg/m^2^** | **<30 kg/m^2^** | | **P for interaction** | |
| HDL-P | | 1.19 (0.92-1.53) | 1.12 (0.91-1.37) | 0.672 | | 1.15 (0.94-1.41) | 1.04 (0.91-1.19) | | 0.151 | |
| VL-HDL-P | | **2.13 (1.76-2.58)** | **1.87 (1.62-2.16)** | 0.304 | | 0.81 (0.62-1.06) | 1.03 (0.92-1.17) | | 0.199 | |
| L-HDL-P | | **2.32 (1.85-2.91)** | **1.88 (1.58-2.23)** | 0.265 | | 0.92 (0.71-1.2) | 1.05 (0.92-1.2) | | 0.644 | |
| M-HDL-P | | **1.49 (1.2-1.85)** | **1.34 (1.13-1.59)** | 0.949 | | 1.09 (0.9-1.31) | 1.06 (0.94-1.19) | | 0.333 | |
| S-HDL-P | | **0.64 (0.51-0.82)** | **0.6 (0.49-0.73)** | 0.739 | | **1.23 (1.02-1.5)** | 1 (0.88-1.13) | | 0.023 | |
| IDL-P | | **1.08 (0.71-1.63)** | 1.09 (0.78-1.51) | 0.728 | | 0.88 (0.64-1.23) | 1.07 (0.86-1.32) | | 0.946 | |
| LDL-P | | **0.45 (0.29-0.7)** | **0.48 (0.34-0.68)** | 0.962 | | 0.84 (0.58-1.24) | 0.91 (0.72-1.14) | | 0.7 | |
| L-LDL-P | | **0.61 (0.4-0.93)** | **0.55 (0.39-0.76)** | 0.854 | | 0.77 (0.55-1.08) | 0.94 (0.76-1.17) | | 0.939 | |
| M-LDL-P | | **0.4 (0.26-0.59)** | **0.51 (0.37-0.7)** | 0.901 | | 1.17 (0.83-1.66) | 0.89 (0.72-1.1) | | 0.289 | |
| S-LDL-P | | **0.47 (0.31-0.72)** | **0.61 (0.43-0.85)** | 0.944 | | 0.91 (0.63-1.32) | 0.89 (0.71-1.12) | | 0.616 | |
| VLDL-P | | **2.16 (1.22-3.8)** | 1.48 (0.94-2.33) | 0.289 | | 0.82 (0.52-1.3) | 1.03 (0.77-1.37) | | 0.762 | |
| CEL-VLDL-P | | 1.17 (0.69-1.98) | **0.56 (0.32-0.96)** | 0.101 | | 1.09 (0.69-1.74) | 1.02 (0.72-1.46) | | 0.679 | |
| VL-VLDL-P | | **0.02 (0.01-0.07)** | **0.02 (0.01-0.05)** | 0.851 | | 1.1 (0.38-3.22) | 0.73 (0.38-1.42) | | 0.618 | |
| L-VLDL-P | | **0.07 (0.03-0.16)** | **0.08 (0.04-0.15)** | 0.747 | | 0.84 (0.39-1.8) | 0.74 (0.45-1.22) | | 0.701 | |
| M-VLDL-P | | **0.45 (0.27-0.73)** | **0.41 (0.28-0.6)** | 0.801 | | 0.88 (0.58-1.33) | 0.92 (0.71-1.19) | | 0.663 | |
| S-VLDL-P | | 1.24 (0.77-2) | 1.14 (0.76-1.69) | 0.355 | | 0.94 (0.63-1.4) | 0.95 (0.74-1.23) | | 0.587 | |
| VS-VLDL-P | | **2.81 (2.21-3.58)** | **2.58 (2.08-3.21)** | 0.798 | | 0.86 (0.65-1.15) | 1.13 (0.94-1.35) | | 0.775 | |

Models were fully adjusted with age, sex, ethnicity, BMI, history of cardiovascular disease (CVD), diabetes mellitus (DM), diet score, alcohol status, smoking status, lipid-lowing drugs, MET, TC, TG and Townsend deprivation index. VL-HDL-P: very large HDL particles; L-HDL-P: large HDL articles; M-HDL-P: medium HDL particles; S-HDL-P: small HDL particles; L-LDL-P: large LDL particles; M-LDL-P: medium LDL particles; S-LDL-P: small LDL particles; CEL-VLDL-P: chylomicrons and extremely large VLDL particles; VL-VLDL-P: very large VLDL particles; L-VLDL-P: large VLDL particles; M-VLDL-P: medium VLDL particles; S-VLDL-P: small VLDL particles; VS-VLDL-P: very small VLDL particles.

eTable 8 Subgroup and interaction analysis between lipoprotein particles and the risk of gastrointestinal cancer across age

| **Type** | **Overall** | |  | **Esophageal** | |  | **Stomach** | |  |
| --- | --- | --- | --- | --- | --- | --- | --- | --- | --- |
|  | **≥60 years** | **<60 years** | **P for interaction** | **≥60 years** | **<60 years** | **P for interaction** | **≥60 years** | **<60 years** | **P for interaction** |
| HDL-P | 1.02 (0.97-1.07) | 1.01 (0.94-1.07) | 0.65 | 1.03 (0.89-1.2) | 1.21 (1-1.47) | 0.214 | 1 (0.83-1.21) | 0.84 (0.64-1.1) | 0.184 |
| VL-HDL-P | **1.07 (1.02-1.12)** | **1.14 (1.07-1.21)** | 0.45 | 1.1 (0.95-1.27) | 1.2 (0.98-1.46) | 0.178 | 0.88 (0.73-1.07) | 0.99 (0.74-1.32) | 0.654 |
| L-HDL-P | **1.07 (1.02-1.13)** | **1.12 (1.05-1.19)** | 0.891 | 1.09 (0.94-1.28) | **1.29 (1.05-1.57)** | 0.216 | 0.91 (0.74-1.11) | 0.9 (0.66-1.23) | 0.219 |
| M-HDL-P | 1.03 (0.99-1.08) | 1.04 (0.98-1.1) | 0.881 | 1.05 (0.92-1.2) | **1.28 (1.08-1.52)** | 0.746 | 0.99 (0.83-1.17) | 0.83 (0.64-1.06) | 0.154 |
| S-HDL-P | 0.97 (0.92-1.02) | **0.92 (0.86-0.98)** | 0.397 | 0.96 (0.83-1.11) | 1 (0.82-1.21) | 0.2 | 1.07 (0.9-1.28) | 0.9 (0.69-1.16) | 0.413 |
| IDL-P | 0.98 (0.9-1.06) | 1.02 (0.92-1.13) | 0.945 | 0.98 (0.77-1.24) | 1.09 (0.79-1.49) | 0.06 | 1.02 (0.76-1.37) | 1.03 (0.67-1.6) | 0.537 |
| LDL-P | 0.93 (0.85-1.02) | 0.9 (0.8-1.01) | 0.993 | 0.89 (0.68-1.17) | **0.58 (0.41-0.82)** | 0.023 | 1.02 (0.73-1.44) | 1.45 (0.87-2.39) | 0.661 |
| L-LDL-P | 0.95 (0.87-1.03) | 0.9 (0.81-1) | 0.792 | 0.89 (0.69-1.13) | **0.6 (0.44-0.83)** | 0.011 | 0.96 (0.71-1.31) | 1.29 (0.82-2.01) | 0.786 |
| M-LDL-P | 0.94 (0.87-1.02) | 0.95 (0.86-1.05) | 0.701 | 0.96 (0.75-1.22) | 0.76 (0.56-1.04) | 0.092 | 1.18 (0.87-1.6) | 1.4 (0.92-2.13) | 0.593 |
| S-LDL-P | 0.94 (0.87-1.03) | 0.96 (0.86-1.08) | 0.622 | 0.95 (0.73-1.23) | **0.6 (0.43-0.84)** | 0.065 | 0.97 (0.7-1.35) | 1.4 (0.86-2.27) | 0.355 |
| VLDL-P | 1.01 (0.9-1.13) | 0.99 (0.86-1.14) | 0.441 | 0.94 (0.67-1.31) | 0.88 (0.57-1.36) | 0.932 | 1.09 (0.72-1.65) | 1.07 (0.6-1.92) | 0.184 |
| CEL-VLDL-P | 1.06 (0.93-1.2) | 1.06 (0.9-1.25) | 0.228 | 1.25 (0.85-1.83) | 1.57 (0.99-2.47) | 0.026 | 0.93 (0.58-1.48) | **0.53 (0.28-0.97)** | 0.041 |
| VL-VLDL-P | **0.74 (0.58-0.96)** | **0.59 (0.42-0.81)** | 0.296 | 0.8 (0.37-1.73) | **0.28 (0.11-0.73)** | 0.115 | 1.55 (0.59-4.08) | 1.87 (0.48-7.25) | 0.019 |
| L-VLDL-P | **0.78 (0.65-0.94)** | **0.6 (0.47-0.76)** | 0.441 | 0.68 (0.39-1.19) | **0.28 (0.15-0.54)** | 0.283 | 1.69 (0.82-3.48) | **2.78 (1.05-7.42)** | 0.016 |
| M-VLDL-P | 0.93 (0.85-1.03) | **0.82 (0.73-0.93)** | 0.976 | 0.84 (0.63-1.12) | **0.58 (0.4-0.83)** | 0.311 | 1.23 (0.85-1.77) | 1.39 (0.83-2.31) | 0.277 |
| S-VLDL-P | 0.99 (0.9-1.09) | 0.91 (0.81-1.04) | 0.55 | 0.89 (0.67-1.19) | 0.71 (0.49-1.04) | 0.848 | 1.06 (0.75-1.52) | 1.07 (0.65-1.77) | 0.12 |
| VS-VLDL-P | 1.07 (1-1.14) | **1.18 (1.08-1.28)** | 0.209 | 1.08 (0.87-1.33) | **1.41 (1.09-1.82)** | 0.935 | 0.95 (0.73-1.24) | 0.85 (0.58-1.24) | 0.878 |

| **Continued eTable 8. Subgroup and interaction analysis between lipoprotein particles and the risk of gastrointestinal cancer across age** | | | | | | |
| --- | --- | --- | --- | --- | --- | --- |
| **Type** | **Small intestine** | |  | **Colorectal** | |  |
|  | **≥60 years** | **<60 years** | **P for interaction** | **≥60 years** | **<60 years** | **P for interaction** |
| HDL-P | 0.79 (0.57-1.08) | 1.29 (0.86-1.95) | 0.112 | 0.98 (0.92-1.05) | 0.98 (0.91-1.07) | 0.286 |
| VL-HDL-P | 0.86 (0.63-1.19) | 1.38 (0.94-2.04) | 0.291 | 1.03 (0.97-1.1) | 1.06 (0.98-1.15) | 0.169 |
| L-HDL-P | 0.77 (0.54-1.09) | 1.47 (0.97-2.21) | 0.24 | 1.02 (0.95-1.09) | 1.04 (0.95-1.13) | 0.249 |
| M-HDL-P | 0.78 (0.59-1.04) | 1.28 (0.89-1.85) | 0.106 | 0.99 (0.93-1.05) | 1 (0.93-1.07) | 0.275 |
| S-HDL-P | 0.93 (0.69-1.25) | 1.04 (0.68-1.57) | 0.273 | 0.97 (0.91-1.03) | 0.95 (0.88-1.03) | 0.642 |
| IDL-P | 1.22 (0.75-1.98) | 0.86 (0.43-1.7) | 0.532 | 0.99 (0.89-1.1) | 0.97 (0.85-1.1) | 0.381 |
| LDL-P | 1.45 (0.82-2.57) | 0.57 (0.27-1.19) | 0.701 | 1.03 (0.91-1.15) | 1 (0.86-1.16) | 0.381 |
| L-LDL-P | 1.2 (0.72-1.98) | **0.5 (0.26-0.98)** | 0.855 | 1.04 (0.93-1.15) | 0.99 (0.87-1.13) | 0.435 |
| M-LDL-P | 1.63 (0.98-2.7) | 0.99 (0.5-1.97) | 0.5 | 0.98 (0.89-1.09) | 1 (0.88-1.14) | 0.348 |
| S-LDL-P | 1.6 (0.92-2.77) | 0.79 (0.38-1.65) | 0.555 | 1.03 (0.91-1.15) | 1.06 (0.92-1.22) | 0.306 |
| VLDL-P | 1.2 (0.61-2.32) | 0.8 (0.32-2.03) | 0.37 | 1.01 (0.86-1.15) | 0.96 (0.8-1.15) | 0.517 |
| CEL-VLDL-P | 1.16 (0.56-2.42) | 1.29 (0.48-3.49) | 0.519 | 1.07 (0.91-1.26) | 1.06 (0.86-1.31) | 0.999 |
| VL-VLDL-P | **1.01 (1.99-51.69)** | 0.19 (0.03-1.38) | 0.633 | 0.99 (0.71-1.39) | 0.79 (0.53-1.19) | 0.962 |
| L-VLDL-P | **4.89 (1.43-16.42)** | 0.29 (0.07-1.21) | 0.684 | 0.99 (0.77-1.26) | **0.74 (0.55-0.99)** | 0.971 |
| M-VLDL-P | 1.63 (0.89-2.98) | 0.61 (0.28-1.34) | 0.655 | 1.01 (0.89-1.15) | 0.89 (0.76-1.04) | 0.748 |
| S-VLDL-P | 1.05 (0.59-1.86) | 0.9 (0.4-2.02) | 0.335 | 1 (0.88-1.13) | 0.93 (0.79-1.09) | 0.72 |
| VS-VLDL-P | 0.88 (0.58-1.35) | 1.1 (0.62-1.96) | 0.193 | 0.99 (0.9-1.08) | 1.06 (0.95-1.19) | 0.133 |

| **Continued eTable 8. Subgroup and interaction analysis between lipoprotein particles and the risk of gastrointestinal cancer across age** | | | | | | |
| --- | --- | --- | --- | --- | --- | --- |
| **Type** | **Liver** | |  | **Pancreatic** | |  |
|  | **≥60 years** | **<60 years** | **P for interaction** | **≥60 years** | **<60 years** | **P for interaction** |
| HDL-P | **1.38 (1.14-1.67)** | 0.84 (0.64-1.09) | 0.004 | 1.08 (0.94-1.23) | 1.08 (0.89-1.3) | 0.802 |
| VL-HDL-P | **1.79 (1.55-2.07)** | **2.11 (1.78-2.5)** | 0.132 | 1.03 (0.9-1.17) | 0.93 (0.76-1.13) | 0.081 |
| L-HDL-P | **1.99 (1.68-2.36)** | **1.99 (1.6-2.48)** | 0.693 | 1.06 (0.92-1.21) | 0.97 (0.79-1.19) | 0.148 |
| M-HDL-P | **1.59 (1.35-1.87)** | 1.13 (0.9-1.42) | 0.014 | 1.08 (0.96-1.22) | 1.05 (0.88-1.24) | 0.566 |
| S-HDL-P | **0.77 (0.63-0.93)** | **0.44 (0.35-0.54)** | <0.001 | 1.03 (0.91-1.18) | 1.12 (0.93-1.35) | 0.387 |
| IDL-P | 0.82 (0.59-1.14) | **1.56 (1.04-2.34)** | 0.638 | 0.96 (0.77-1.19) | 1.09 (0.8-1.49) | 0.797 |
| LDL-P | **0.39 (0.28-0.55)** | **0.62 (0.4-0.98)** | 0.919 | 0.88 (0.69-1.11) | 0.91 (0.64-1.29) | 0.839 |
| L-LDL-P | **0.48 (0.35-0.67)** | 0.7 (0.46-1.07) | 0.924 | 0.89 (0.72-1.11) | 0.89 (0.65-1.22) | 0.7 |
| M-LDL-P | **0.4 (0.29-0.54)** | **0.59 (0.39-0.88)** | 0.869 | 0.93 (0.75-1.15) | 1.02 (0.74-1.4) | 0.909 |
| S-LDL-P | **0.45 (0.32-0.61)** | 0.78 (0.5-1.21) | 0.899 | 0.87 (0.69-1.1) | 0.95 (0.68-1.33) | 0.988 |
| VLDL-P | 1.28 (0.82-1.99) | **2.37 (1.33-4.2)** | 0.367 | 0.98 (0.73-1.31) | 0.91 (0.6-1.4) | 0.993 |
| CEL-VLDL-P | 0.87 (0.55-1.38) | 0.75 (0.4-1.44) | 0.133 | 1.01 (0.71-1.42) | 1.15 (0.71-1.88) | 0.5 |
| VL-VLDL-P | **0.02 (0.01-0.04)** | **0.04 (0.01-0.11)** | 0.166 | 0.68 (0.35-1.35) | 1.18 (0.44-3.17) | 0.487 |
| L-VLDL-P | **0.07 (0.04-0.13)** | **0.42 (0.04-0.21)** | 0.137 | 0.69 (0.42-1.15) | 0.94 (0.46-1.93) | 0.529 |
| M-VLDL-P | **0.37 (0.26-0.54)** | **0.5 (0.31-0.81)** | 0.388 | 0.9 (0.69-1.17) | 0.93 (0.64-1.35) | 0.957 |
| S-VLDL-P | 1.09 (0.75-1.58) | 1.17 (0.71-1.94) | 0.176 | 0.95 (0.74-1.23) | 0.92 (0.63-1.35) | 0.819 |
| VS-VLDL-P | **2.35 (1.86-2.96)** | **2.94 (2.37-3.63)** | 0.859 | 1.07 (0.89-1.29) | 0.96 (0.73-1.26) | 0.487 |

Models were fully adjusted with age, sex, ethnicity, BMI, history of cardiovascular disease (CVD), diabetes mellitus (DM), diet score, alcohol status, smoking status, lipid-lowing drugs, MET, TC, TG and Townsend deprivation index. VL-HDL-P: very large HDL particles; L-HDL-P: large HDL articles; M-HDL-P: medium HDL particles; S-HDL-P: small HDL particles; L-LDL-P: large LDL particles; M-LDL-P: medium LDL particles; S-LDL-P: small LDL particles; CEL-VLDL-P: chylomicrons and extremely large VLDL particles; VL-VLDL-P: very large VLDL particles; L-VLDL-P: large VLDL particles; M-VLDL-P: medium VLDL particles; S-VLDL-P: small VLDL particles; VS-VLDL-P: very small VLDL particles.

| eTable 9. Sensitivity analysis between lipoprotein particles and the risk of gastrointestinal cancer with participants exclude gastrointestinal cancer occurred within 2 years | | | | | | | |
| --- | --- | --- | --- | --- | --- | --- | --- |
| **Type** | **Overall** | **Esophageal** | **Stomach** | **Small intestine** | **Colorectal** | **Liver** | **Pancreatic** |
| HDL-P | 1.01 (0.97-1.06) | 1.12 (0.99-1.27) | 0.95 (0.81-1.12) | 0.95 (0.73-1.23) | 0.97 (0.92-1.03) | **1.22 (1.03-1.44)** | 1.06 (0.94-1.19) |
| VL-HDL-P | **1.09 (1.04-1.13)** | **1.14 (1.01-1.29)** | 0.85 (0.71-1.02) | 0.99 (0.76-1.29) | 1.04 (0.99-1.1) | **1.93 (1.71-2.17)** | 0.99 (0.88-1.11) |
| L-HDL-P | **1.08 (1.03-1.13)** | **1.17 (1.03-1.33)** | 0.86 (0.72-1.03) | 0.95 (0.72-1.26) | 1.02 (0.96-1.08) | **2.04 (1.77-2.36)** | 1.02 (0.9-1.15) |
| M-HDL-P | 1.03 (1-1.07) | **1.15 (1.03-1.29)** | 0.93 (0.8-1.08) | 0.93 (0.74-1.18) | 0.98 (0.94-1.03) | **1.48 (1.28-1.7)** | 1.05 (0.95-1.17) |
| S-HDL-P | **0.95 (0.92-0.99)** | 0.99 (0.88-1.12) | 1.05 (0.9-1.23) | 0.99 (0.77-1.27) | 0.96 (0.91-1.01) | **0.63 (0.54-0.74)** | 1.05 (0.94-1.17) |
| IDL-P | 1.02 (0.95-1.09) | 1.01 (0.82-1.23) | 1.05 (0.81-1.37) | 1.06 (0.7-1.61) | 1.01 (0.93-1.1) | 1.01 (0.77-1.33) | 1.04 (0.87-1.26) |
| LDL-P | **0.92 (0.85-0.99)** | **0.73 (0.58-0.91)** | 1.14 (0.84-1.54) | 0.95 (0.66-1.69) | 1.02 (0.93-1.13) | **0.43 (0.32-0.57)** | 0.9 (0.73-1.11) |
| L-LDL-P | **0.92 (0.86-0.99)** | **0.73 (0.59-0.9)** | 1.03 (0.78-1.35) | 0.88 (0.579-1.34) | 1.02 (0.93-1.12) | **0.53 (0.4-0.69)** | 0.9 (0.74-1.09) |
| M-LDL-P | 0.94 (0.88-1.01) | 0.87 (0.71-1.06) | 1.31 (1-1.72) | 1.39 (0.92-2.12) | 1 (0.91-1.09) | **0.42 (0.32-0.55)** | 0.96 (0.8-1.17) |
| S-LDL-P | 0.96 (0.89-1.03) | **0.79 (0.63-0.98)** | 1.12 (0.83-1.5) | 1.29 (0.81-2.05) | 1.05 (0.95-1.16) | **0.49 (0.38-0.65)** | 0.91 (0.74-1.11) |
| VLDL-P | 1 (0.91-1.09) | 0.87 (0.66-1.15) | 1.07 (0.74-1.53) | 1 (0.57-1.76) | 0.98 (0.87-1.11) | **1.59 (1.1-2.32)** | 0.94 (0.73-1.22) |
| CEL-VLDL-P | 1.1 (0.99-1.23) | **1.59 (1.17-2.17)** | 0.78 (0.51-1.18) | 1.25 (0.68-2.29) | 1.11 (0.96-1.27) | 0.8 (0.54-1.19) | 1.05 (0.78-1.41) |
| VL-VLDL-P | **0.72 (0.58-0.9)** | 0.56 (0.3-1.06) | 2.13 (0.9-5.03) | 2.73 (0.72-10.32) | 0.97 (0.74-1.28) | **0.02 (0-0.04)** | 0.92 (0.51-1.67) |
| L-VLDL-P | **0.7 (0.6-0.82)** | **0.42 (0.27-0.66)** | **2.32 (1.22-4.43)** | 1.63 (0.62-4.27) | 0.87 (0.71-1.07) | **0.07 (0.04-0.11)** | 0.82 (0.53-1.27) |
| M-VLDL-P | **0.88 (0.81-0.96)** | **0.68 (0.54-0.87)** | 1.31 (0.94-1.81) | 1.13 (0.69-1.86) | 0.96 (0.86-1.06) | **0.39 (0.29-0.53)** | 0.92 (0.73-1.15) |
| S-VLDL-P | 0.94 (0.87-1.03) | **0.78 (0.61-1)** | 1.05 (0.76-1.44) | 0.98 (0.6-1.58) | 0.96 (0.86-1.07) | 1.14 (0.83-1.57) | 0.91 (0.73-1.14) |
| VS-VLDL-P | **1.11 (1.04-1.17)** | 1.18 (0.99-1.41) | 0.89 (0.7-1.12) | 0.92 (0.64-1.31) | 1.02 (0.95-1.1) | **2.69 (2.26-3.19)** | 1.02 (0.87-1.2) |

Models were fully adjusted with age, sex, ethnicity, BMI, history of cardiovascular disease (CVD), diabetes mellitus (DM), diet score, alcohol status, smoking status, lipid-lowing drugs, MET, TC, TG and Townsend deprivation index. VL-HDL-P: very large HDL particles; L-HDL-P: large HDL articles; M-HDL-P: medium HDL particles; S-HDL-P: small HDL particles; L-LDL-P: large LDL particles; M-LDL-P: medium LDL particles; S-LDL-P: small LDL particles; CEL-VLDL-P: chylomicrons and extremely large VLDL particles; VL-VLDL-P: very large VLDL particles; L-VLDL-P: large VLDL particles; M-VLDL-P: medium VLDL particles; S-VLDL-P: small VLDL particles; VS-VLDL-P: very small VLDL particles.

| eTable 10. Sensitivity analysis between lipoprotein particles and the risk of gastrointestinal cancer with participants exclude all missing values at baseline | | | | | | | |
| --- | --- | --- | --- | --- | --- | --- | --- |
| **Type** | **Overall** | **Esophageal** | **Stomach** | **Small intestine** | **Colorectal** | **Liver** | **Pancreatic** |
| HDL-P | 0.99 (0.95-1.04) | 1.14 (0.99-1.31) | 0.93 (0.78-1.11) | 0.85 (0.62-1.15) | 0.97 (0.92-1.03) | 1.11 (0.93-1.33) | 1.06 (0.94-1.21) |
| VL-HDL-P | **1.08 (1.03-1.14)** | 1.14 (0.99-1.31) | 0.85 (0.7-1.03) | 0.94 (0.69-1.27) | 1.02 (0.97-1.08) | **1.91 (1.68-2.17)** | 0.96 (0.85-1.09) |
| L-HDL-P | **1.07 (1.01-1.12)** | **1.19 (1.03-1.38)** | 0.85 (0.7-1.03) | 0.85 (0.62-1.18) | 1 (0.94-1.06) | **1.98 (1.7-2.31)** | 0.99 (0.87-1.13) |
| M-HDL-P | 1.01 (0.97-1.06) | **1.17 (1.04-1.33)** | 0.92 (0.78-1.07) | 0.83 (0.63-1.09) | 0.97 (0.92-1.02) | **1.36 (1.16-1.59)** | 1.05 (0.94-1.17) |
| S-HDL-P | **0.94 (0.9-0.99)** | 1 (0.87-1.15) | 1.03 (0.87-1.22) | 0.95 (0.71-1.27) | 0.97 (0.92-1.03) | **0.58 (0.49-0.69)** | 1.07 (0.95-1.21) |
| IDL-P | 1 (0.93-1.08) | 0.98 (0.78-1.22) | 1.15 (0.87-1.51) | 1.13 (0.71-1.81) | 0.98 (0.9-1.08) | 1.07 (0.8-1.44) | 1.01 (0.82-1.23) |
| LDL-P | 0.96 (0.88-1.05) | **0.7 (0.55-0.9)** | 1.16 (0.84-1.6) | 1.29 (0.75-2.23) | 1.06 (0.95-1.18) | **0.49 (0.36-0.68)** | 0.92 (0.74-1.16) |
| L-LDL-P | 0.96 (0.88-1.04) | **0.72 (0.57-0.9)** | 1.02 (0.77-1.35) | 1.04 (0.64-1.69) | 1.04 (0.95-1.15) | **0.61 (0.45-0.82)** | 0.92 (0.75-1.13) |
| M-LDL-P | 0.99 (0.91-1.07) | 0.83 (0.66-1.05) | **1.41 (1.06-1.87)** | 1.59 (0.98-2.58) | 1.04 (0.95-1.15) | **0.45 (0.34-0.6)** | 0.99 (0.8-1.21) |
| S-LDL-P | 1 (0.91-1.09) | **0.73 (0.57-0.93)** | 1.14 (0.84-1.55) | 1.53 (0.9-2.61) | 1.08 (0.98-1.2) | **0.57 (0.42-0.77)** | 0.92 (0.74-1.15) |
| VLDL-P | 1.03 (0.93-1.15) | 0.84 (0.61-1.15) | 1.14 (0.78-1.68) | 0.98 (0.52-1.87) | 1.02 (0.9-1.16) | **1.59 (1.06-2.39)** | 0.97 (0.73-1.28) |
| CEL-VLDL-P | 1.05 (0.93-1.19) | 1.37 (0.97-1.94) | 0.88 (0.58-1.36) | 1.12 (0.55-2.28) | 1.04 (0.9-1.2) | 0.88 (0.56-1.36) | 1.12 (0.81-1.54) |
| VL-VLDL-P | **0.75 (0.59-0.96)** | **0.44 (0.22-0.88)** | 1.96 (0.8-4.83) | **5.89 (1.24-27.94)** | 1.01 (0.75-1.36) | **0.03 (0.01-0.07)** | 0.9 (0.47-1.72) |
| L-VLDL-P | **0.78 (0.65-0.93)** | **0.4 (0.25-0.67)** | **1.99 (1.02-3.87)** | **3.45 (1.09-10.99)** | 0.98 (0.79-1.22) | **0.09 (0.05-0.16)** | 0.78 (0.49-1.26) |
| M-VLDL-P | 0.93 (0.85-1.03) | **0.67 (0.51-0.88)** | 1.3 (0.92-1.82) | 1.37 (0.77-2.43) | 1.02 (0.91-1.14) | **0.44 (0.31-0.62)** | 0.93 (0.73-1.19) |
| S-VLDL-P | 0.99 (0.91-1.09) | 0.78 (0.6-1.02) | 1.08 (0.78-1.51) | 0.94 (0.54-1.63) | 1.02 (0.91-1.14) | 1.1 (0.77-1.56) | 0.98 (0.77-1.25) |
| VS-VLDL-P | **1.1 (1.03-1.18)** | 1.16 (0.95-1.41) | 0.96 (0.76-1.23) | 0.8 (0.53-1.21) | 1.01 (0.93-1.09) | **2.57 (2.11-3.12)** | 1.02 (0.86-1.22) |

Models were fully adjusted with age, sex, ethnicity, BMI, history of cardiovascular disease (CVD), diabetes mellitus (DM), diet score, alcohol status, smoking status, lipid-lowing drugs, MET, TC, TG and Townsend deprivation index. VL-HDL-P: very large HDL particles; L-HDL-P: large HDL articles; M-HDL-P: medium HDL particles; S-HDL-P: small HDL particles; L-LDL-P: large LDL particles; M-LDL-P: medium LDL particles; S-LDL-P: small LDL particles; CEL-VLDL-P: chylomicrons and extremely large VLDL particles; VL-VLDL-P: very large VLDL particles; L-VLDL-P: large VLDL particles; M-VLDL-P: medium VLDL particles; S-VLDL-P: small VLDL particles; VS-VLDL-P: very small VLDL particles.

| eTable 11. Sensitivity analysis between lipoprotein particles and the risk of gastrointestinal cancer with random forest imputation | | | | | | | |
| --- | --- | --- | --- | --- | --- | --- | --- |
| **Type** | **Overall** | **Esophageal** | **Stomach** | **Small intestine** | **Colorectal** | **Liver** | **Pancreatic** |
| HDL-P | 1.02 (0.98-1.06) | 1.1 (0.97-1.23) | 0.95 (0.81-1.11) | 0.93 (0.73-1.2) | 0.98 (0.94-1.04) | 1.16 (0.99-1.36) | 1.08 (0.96-1.2) |
| VL-HDL-P | **1.1 (1.06-1.14)** | **1.15 (1.02-1.29)** | 0.94 (0.8-1.1) | 1.02 (0.8-1.31) | 1.05 (1-1.1) | **1.91 (1.7-2.13)** | 1 (0.9-1.11) |
| L-HDL-P | **1.09 (1.05-1.13)** | **1.17 (1.04-1.32)** | 0.93 (0.79-1.1) | 0.97 (0.74-1.27) | 1.03 (0.98-1.08) | **1.98 (1.73-2.26)** | 1.03 (0.92-1.15) |
| M-HDL-P | 1.04 (1-1.07) | **1.14 (1.02-1.26)** | 0.94 (0.82-1.08) | 0.93 (0.74-1.17) | 0.99 (0.95-1.04) | **1.41 (1.23-1.61)** | 1.07 (0.97-1.18) |
| S-HDL-P | **0.95 (0.91-0.99)** | 0.97 (0.86-1.09) | 1.01 (0.87-1.16) | 0.95 (0.75-1.21) | 0.96 (0.92-1.01) | **0.62 (0.53-0.72)** | 1.06 (0.95-1.18) |
| IDL-P | 0.99 (0.93-1.06) | 1.02 (0.84-1.23) | 1.01 (0.79-1.29) | 1.08 (0.73-1.61) | 0.98 (0.9-1.06) | 106 (0.82-1.37) | 1 (0.84-1.2) |
| LDL-P | **0.92 (0.86-0.99)** | **0.75 (0.61-0.93)** | 1.12 (0.84-1.48) | 1.06 (0.67-1.66) | 1.02 (0.93-1.11) | **0.46 (0.35-0.6)** | 0.89 (0.73-1.08) |
| L-LDL-P | **0.93 (0.87-0.99)** | **0.76 (0.63-0.92)** | 1.03 (0.8-1.33) | 0.89 (0.6-1.34) | 1.02 (0.94-1.11) | **0.55 (0.43-0.72)** | 0.89 (0.74-1.06) |
| M-LDL-P | 0.94 (0.88-1.01) | 0.87 (0.72-1.06) | 1.24 (0.97-1.59) | 1.38 (0.92-2.07) | 0.99 (0.91-1.07) | **0.46 (0.36-0.59)** | 0.95 (0.8-1.14) |
| S-LDL-P | 0.95 (0.89-1.02) | **0.79 (0.65-0.98)** | 1.08 (0.82-1.41) | 1.26 (0.81-1.97) | 1.04 (0.95-1.13) | **0.54 (0.42-0.7)** | 0.89 (0.74-1.08) |
| VLDL-P | 1 (0.92-1.09) | 0.9 (0.69-1.17) | 1.05 (0.75-1.47) | 1.05 (0.61-1.81) | 0.98 (0.88-1.1) | **1.59 (1.12-2.26)** | 0.95 (0.75-1.22) |
| CEL-VLDL-P | 1.06 (0.96-1.17) | **1.4 (1.04-1.87)** | 0.77 (0.53-1.12) | 1.2 (0.67-2.17) | 1.06 (0.93-1.21) | 0.83 (0.57-1.21) | 1.06 (0.8-1.4) |
| VL-VLDL-P | **0.68 (0.56-0.83)** | 0.52 (0.28-0.94) | 1.57 (0.72-3.43) | 2.33 (0.65-8.37) | 0.91 (0.7-1.17) | **0.02 (0.01-0.05)** | 0.81 (0.46-1.42) |
| L-VLDL-P | **0.71 (0.61-0.82)** | **0.45 (0.3-0.69)** | **1.9 (1.06-3.39)** | 1.65 (0.65-4.2) | 0.88 (0.73-1.06) | **0.08 (0.05-0.12)** | 0.76 (0.51-1.15) |
| M-VLDL-P | **0.9 (0.82-0.96)** | **0.71 (0.57-0.89)** | 1.24 (0.92-1.67) | 1.16 (0.72-1.87) | 0.96 (0.87-1.06) | **0.41 (0.31-0.55)** | 0.9 (0.73-1.12) |
| S-VLDL-P | 0.96 (0.89-1.03) | 0.8 (0.64-1.01) | 1.04 (0.78-1.39) | 1 (0.63-1.6) | 0.97 (0.88-1.07) | 1.11 (0.82-1.5) | 0.94 (0.76-1.16) |
| VS-VLDL-P | **1.11 (1.05-1.17)** | **1.2 (1.02-1.41)** | 0.91 (0.74-1.13) | 0.96 (0.68-1.35) | 1.01 (0.94-1.09) | **2.62 (2.23-3.07)** | 1.04 (0.89-1.21) |

Models were fully adjusted with age, sex, ethnicity, BMI, history of cardiovascular disease (CVD), diabetes mellitus (DM), diet score, alcohol status, smoking status, lipid-lowing drugs, MET, TC, TG and Townsend deprivation index. VL-HDL-P: very large HDL particles; L-HDL-P: large HDL articles; M-HDL-P: medium HDL particles; S-HDL-P: small HDL particles; L-LDL-P: large LDL particles; M-LDL-P: medium LDL particles; S-LDL-P: small LDL particles; CEL-VLDL-P: chylomicrons and extremely large VLDL particles; VL-VLDL-P: very large VLDL particles; L-VLDL-P: large VLDL particles; M-VLDL-P: medium VLDL particles; S-VLDL-P: small VLDL particles; VS-VLDL-P: very small VLDL particles

| eTable 12. Sensitivity analysis between lipoprotein particles and the risk of gastrointestinal cancer after further adjusted menopausal status and history of proton pump inhibitor use. | | | | | | | |
| --- | --- | --- | --- | --- | --- | --- | --- |
| **Type** | **Overall** | **Esophageal** | **Stomach** | **Small intestine** | **Colorectal** | **Liver** | **Pancreatic** |
| HDL-P | 1.01 (0.97-1.05) | 1.1 (0.97-1.23) | 0.94 (0.8-1.09) | 0.93 (0.72-1.2) | 0.98 (0.94-1.04) | 1.16 (1-1.36) | 1.06 (0.95-1.18) |
| VL-HDL-P | **1.1 (1.06-1.14)** | **1.16 (1.03-1.3)** | 0.94 (0.8-1.1) | 1.03 (0.8-1.31) | 1.04 (0.99-1.09) | **1.92 (1.72-2.14)** | 1 (0.9-1.11) |
| L-HDL-P | **1.09 (1.04-1.13)** | **1.18 (1.05-1.34)** | 0.93 (0.78-1.1) | 0.97 (0.74-1.27) | 1.03 (0.97-1.08) | **2 (1.75-2.28)** | 1.02 (0.91-1.15) |
| M-HDL-P | 1.03 (1-1.07) | **1.14 (1.02-1.26)** | 0.93 (0.81-1.07) | 0.93 (0.74-1.16) | 0.99 (0.95-1.04) | **1.41 (1.23-1.61)** | 1.06 (0.96-1.16) |
| S-HDL-P | **0.95 (0.91-0.98)** | 0.96 (0.86-1.08) | 0.99 (0.85-1.15) | 0.95 (0.75-1.21) | 0.97 (0.92-1.01) | **0.61 (0.53-0.72)** | 1.05 (0.94-1.17) |
| IDL-P | 1 (0.94-1.06) | 1.01 (0.83-1.22) | 1.03 (0.81-1.31) | 1.09 (0.73-1.61) | 0.98 (0.91-1.06) | 1.04 (0.81-1.35) | 1.02 (0.85-1.22) |
| LDL-P | **0.92 (0.86-0.99)** | **0.75 (0.61-0.93)** | 1.14 (0.86-1.51) | 1.06 (0.67-1.66) | 1.02 (0.93-1.11) | **0.46 (0.35-0.6)** | 0.91 (0.74-1.1) |
| L-LDL-P | **0.93 (0.87-0.99)** | **0.76 (0.63-0.92)** | 1.06 (0.82-1.36) | 0.9 (0.6-1.34) | 1.02 (0.94-1.11) | **0.55 (0.43-0.72)** | 0.91 (0.76-1.08) |
| M-LDL-P | 0.95 (0.89-1.01) | 0.86 (0.71-1.05) | 1.25 (0.98-1.61) | 1.38 (0.92-2.07) | 0.99 (0.91-1.08) | **0.45 (0.36-0.58)** | 0.97 (0.81-1.16) |
| S-LDL-P | 0.96 (0.89-1.03) | **0.79 (0.65-0.97)** | 1.1 (0.84-1.44) | 1.27 (0.81-1.97) | 1.04 (0.95-1.14) | **0.54 (0.42-0.7)** | 0.91 (0.75-1.11) |
| VLDL-P | 1.01 (0.92-1.1) | 0.9 (0.69-1.17) | 1.09 (0.78-1.52) | 1.05 (0.62-1.81) | 0.98 (0.88-1.1) | **1.57 (1.11-2.23)** | 0.98 (0.77-1.25) |
| CEL-VLDL-P | 1.06 (0.96-1.17) | **1.39 (1.04-1.86)** | 0.76 (0.53-1.11) | 1.21 (0.67-2.18) | 1.07 (0.94-1.22) | 0.83 (0.57-1.2) | 1.06 (0.8-1.4) |
| VL-VLDL-P | **0.68 (0.56-0.83)** | **0.5 (0.28-0.91)** | 1.6 (0.73-3.5) | 2.34 (0.65-8.4) | 0.92 (0.71-1.19) | **0.02 (0.01-0.04)** | 0.84 (0.48-1.47) |
| L-VLDL-P | **0.71 (0.61-0.82)** | **0.45 (0.29-0.68)** | **1.94 (1.09-3.47)** | 1.64 (0.65-4.16) | 0.88 (0.73-1.06) | **0.07 (0.05-0.12)** | 0.78 (0.52-1.18) |
| M-VLDL-P | **0.89 (0.83-0.96)** | **0.71 (0.56-0.89)** | 1.27 (0.94-1.71) | 1.15 (0.72-1.86) | 0.96 (0.87-1.06) | **0.41 (0.31-0.55)** | 0.92 (0.74-1.14) |
| S-VLDL-P | 0.96 (0.89-1.04) | 0.8 (0.64-1.01) | 1.06 (0.79-1.41) | 1 (0.63-1.6) | 0.97 (0.88-1.07) | 1.1 (0.82-1.49) | 0.96 (0.77-1.18) |
| VS-VLDL-P | **1.11 (1.05-1.17)** | **1.2 (1.02-1.41)** | 0.93 (0.75-1.15) | 0.96 (0.68-1.35) | 1.01 (0.95-1.09) | **2.62 (2.23-3.08)** | 1.05 (0.9-1.23) |

Models were fully adjusted with age, sex, ethnicity, BMI, history of cardiovascular disease (CVD), diabetes mellitus (DM), diet score, alcohol status, smoking status, lipid-lowing drugs, MET, TC, TG, Townsend deprivation index, menopausal status and history of proton pump inhibitor use. VL-HDL-P: very large HDL particles; L-HDL-P: large HDL articles; M-HDL-P: medium HDL particles; S-HDL-P: small HDL particles; L-LDL-P: large LDL particles; M-LDL-P: medium LDL particles; S-LDL-P: small LDL particles; CEL-VLDL-P: chylomicrons and extremely large VLDL particles; VL-VLDL-P: very large VLDL particles; L-VLDL-P: large VLDL particles; M-VLDL-P: medium VLDL particles; S-VLDL-P: small VLDL particles; VS-VLDL-P: very small VLDL particles

eTable 13. The association between lipoprotein particles and the risk of gastrointestinal cancer in esophageal cancer and liver cancer subtypes.

| **Type** | **Esophageal cancer** | | **Liver cancer** | |
| --- | --- | --- | --- | --- |
|  | **EAC** | **ESCC** | **HCC** | **CAC** |
| HDL-P | 1 (0.86-1.16) | **1.3 (1.05-1.61)** | 1.03 (0.81-1.29) | **1.39 (1.11-1.73)** |
| VL-HDL-P | 1.11 (0.95-1.29) | 1.18 (0.98-1.43) | **2.59 (2.28-2.95)** | 1.19 (0.97-1.47) |
| L-HDL-P | 1.1 (0.94-1.3) | **1.27 (1.04-1.56)** | **2.81 (2.36-3.35)** | **1.33 (1.07-1.65)** |
| M-HDL-P | 1.06 (0.93-1.2) | **1.31 (1.08-1.58)** | **1.5 (1.24-1.82)** | **1.37 (1.13-1.67)** |
| S-HDL-P | 0.91 (0.79-1.05) | 1.08 (0.88-1.34) | **0.39 (0.32-0.48)** | 1.16 (0.93-1.45) |
| IDL-P | 1.13 (0.89-1.44) | 0.82 (0.58-1.18) | **1.53 (1.05-2.23)** | **0.66 (0.45-0.97)** |
| LDL-P | 0.89 (0.68-1.17) | **0.55 (0.38-0.8)** | **0.38 (0.26-0.56)** | **0.53 (0.36-0.78)** |
| L-LDL-P | 0.89 (0.7-1.13) | **0.57 (0.4-0.82)** | **0.53 (0.36-0.78)** | **0.56 (0.39-0.82)** |
| M-LDL-P | 0.97 (0.77-1.24) | **0.69 (0.48-0.98)** | **0.29 (0.2-0.41)** | **0.66 (0.46-0.94)** |
| S-LDL-P | 0.93 (0.72-1.2) | **0.58 (0.4-0.83)** | **0.47 (0.32-0.69)** | **0.57 (0.39-0.83)** |
| VLDL-P | 1.25 (0.9-1.74) | **0.48 (0.3-0.79)** | **4.09 (2.47-6.8)** | **0.56 (0.34-0.93)** |
| CEL-VLDL-P | 1.04 (0.74-1.47) | **4.2 (2.31-7.61)** | 0.78 (0.46-1.33) | 0.81 (0.46-1.42) |
| VL-VLDL-P | 0.48 (0.23-1) | 0.86 (0.28-2.7) | **0 (0-0.01)** | **0.17 (0.06-0.51)** |
| L-VLDL-P | **0.57 (0.34-0.96)** | **0.31 (0.14-0.7)** | **0.02 (0.01-0.04)** | **0.3 (0.14-0.66)** |
| M-VLDL-P | 0.88 (0.67-1.17) | **0.5 (0.33-0.75)** | **0.3 (0.2-0.46)** | **0.49 (0.32-0.75)** |
| S-VLDL-P | 1.08 (0.81-1.42) | **0.41 (0.26-0.64)** | **1.76 (1.14-2.73)** | 0.67 (0.43-1.03) |
| VS-VLDL-P | **1.41 (1.16-1.71)** | 0.84 (0.61-1.15) | **4.18 (3.56-4.91)** | 0.9 (0.65-1.24) |

Models were fully adjusted with age, sex, ethnicity, BMI, history of cardiovascular disease (CVD), diabetes mellitus (DM), diet score, alcohol status, smoking status, lipid-lowing drugs, MET, TC, TG, Townsend deprivation index, menopausal status and history of proton pump inhibitor use. VL-HDL-P: very large HDL particles; L-HDL-P: large HDL articles; M-HDL-P: medium HDL particles; S-HDL-P: small HDL particles; L-LDL-P: large LDL particles; M-LDL-P: medium LDL particles; S-LDL-P: small LDL particles; CEL-VLDL-P: chylomicrons and extremely large VLDL particles; VL-VLDL-P: very large VLDL particles; L-VLDL-P: large VLDL particles; M-VLDL-P: medium VLDL particles; S-VLDL-P: small VLDL particles; VS-VLDL-P: very small VLDL particles


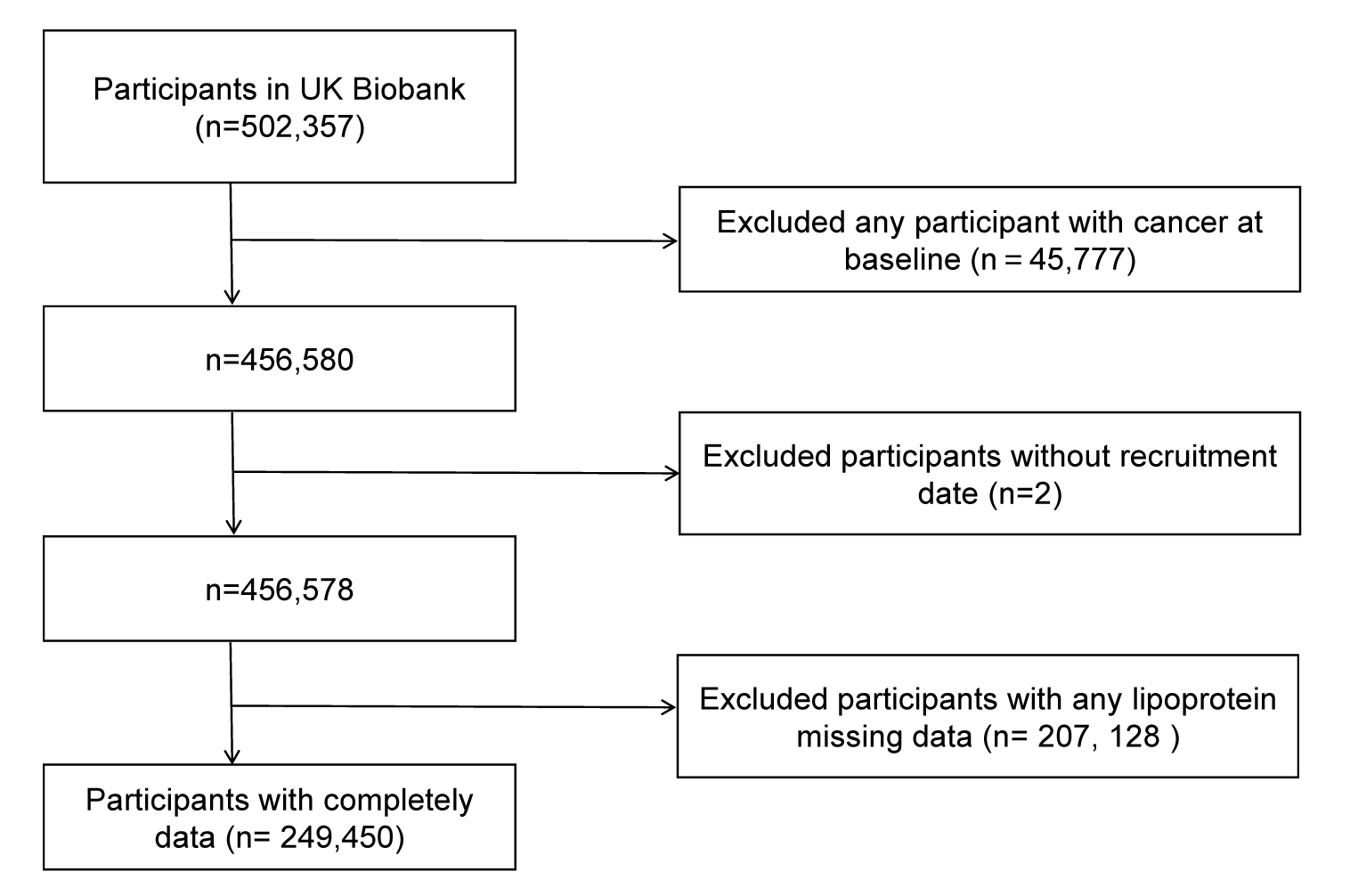


eFigure 1. Study Flowchart


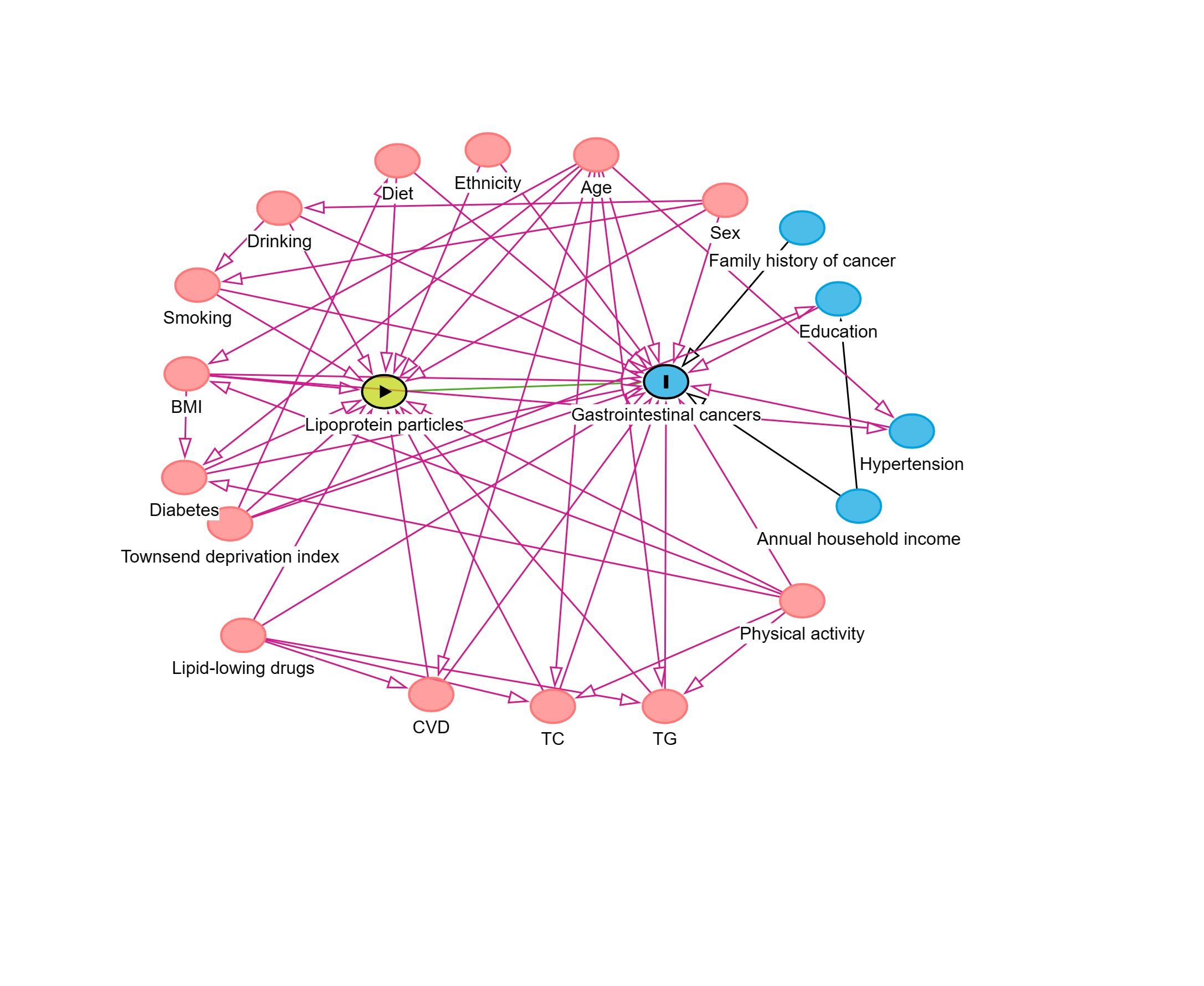


eFigure 2. Directed Acyclic Graph for Covariate Selection

BMI: body mass index; CVD: cardiovascular disease; TC: total cholesterol; TG: triglycerides;


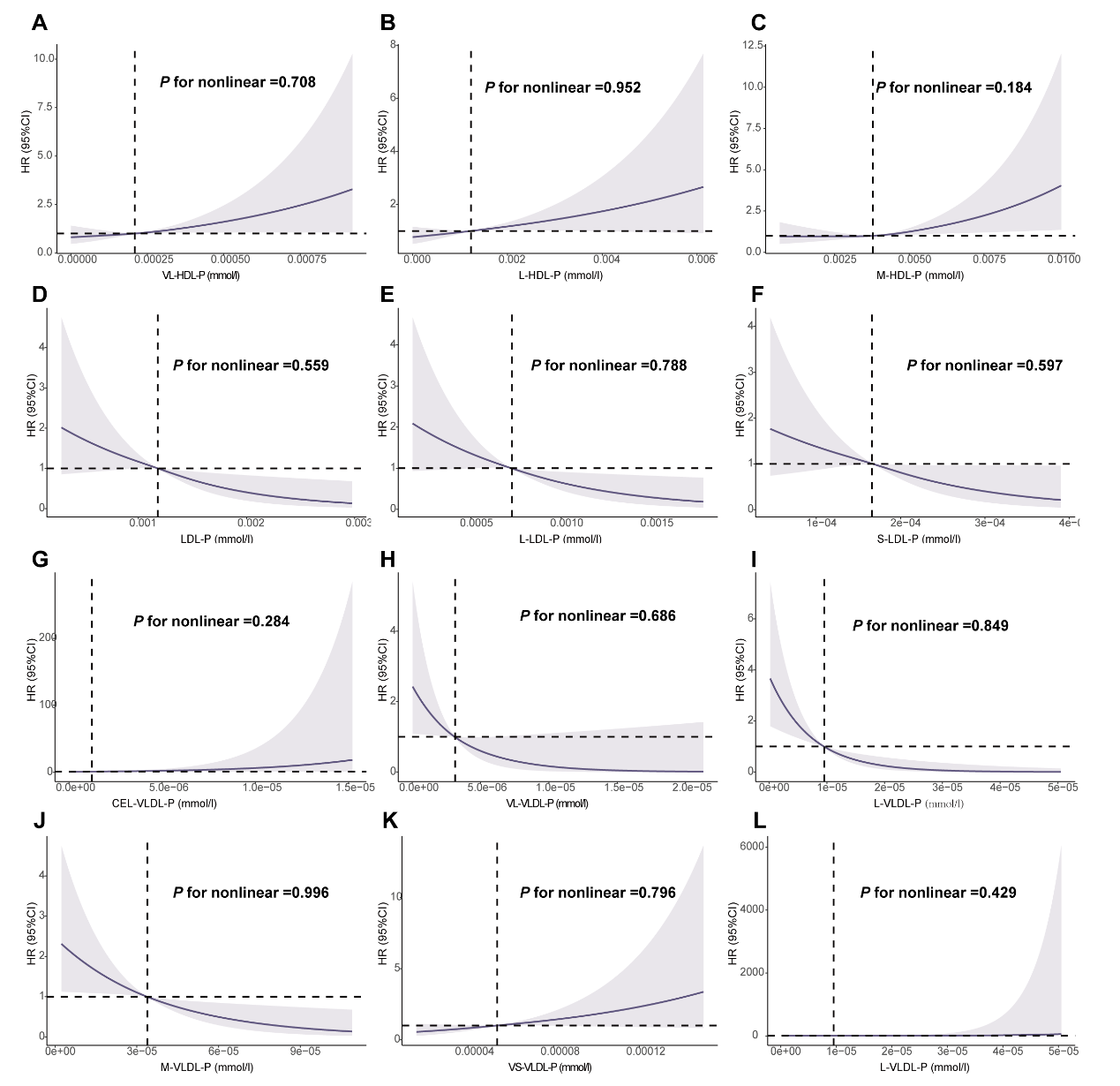


eFigure 3. Association of the lipoprotein particles with esophageal and stomach cancer risk using RCS with 3 knots

A-K. Esophageal cancer. L, stomach cancer. RCS: restricted cubic spline. Models were fully adjusted with age, sex, ethnicity, BMI, history of cardiovascular disease (CVD), diabetes mellitus (DM), diet score, alcohol status, smoking status, lipid-lowing drugs, MET, TC, TG and Townsend deprivation index. VL-HDL-P: very large HDL particles; L-HDL-P: large HDL articles; M-HDL-P: medium HDL particles; L-LDL-P: large LDL particles; S-LDL-P: small LDL particles; CEL-VLDL-P: chylomicrons and extremely large VLDL particles; VL-VLDL-P: very large VLDL particles; L-VLDL-P: large VLDL particles; M-VLDL-P: medium VLDL particles; VS-VLDL-P: very small VLDL particles.


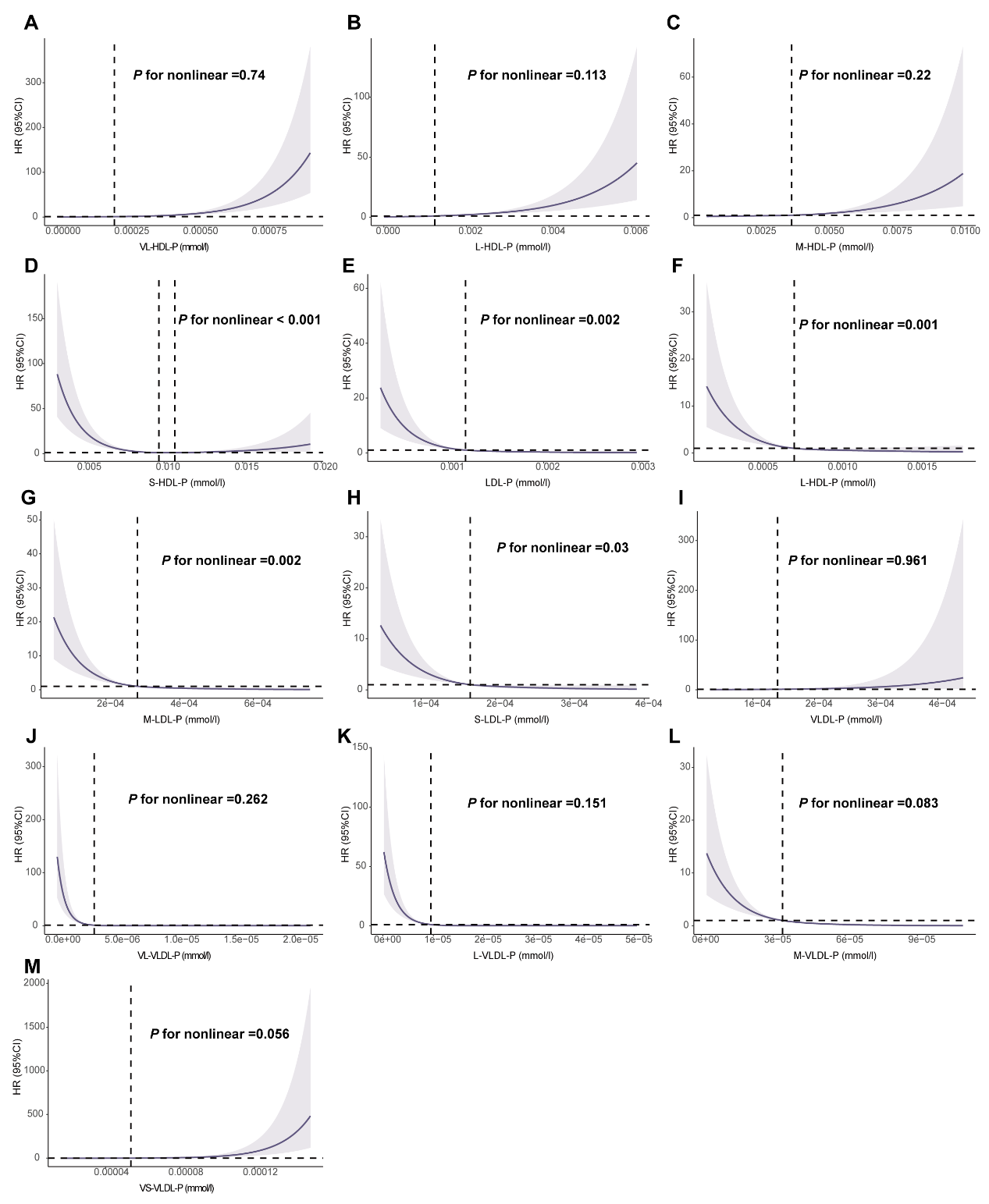


eFigure 4. Association of the lipoprotein particles with liver cancer risk using RCS with 3 knots

A-K. Esophageal cancer. L, stomach cancer. RCS: restricted cubic spline. Models were fully adjusted with age, sex, ethnicity, BMI, history of cardiovascular disease (CVD), diabetes mellitus (DM), diet score, alcohol status, smoking status, lipid-lowing drugs, MET, TC, TG and Townsend deprivation index. VL-HDL-P: very large HDL particles; L-HDL-P: large HDL articles; M-HDL-P: medium HDL particles; S-HDL-P: small HDL particles; L-LDL-P: large LDL particles; M-LDL-P: medium LDL particles; S-LDL-P: small LDL particles; VL-VLDL-P: very large VLDL particles; L-VLDL-P: large VLDL particles; M-VLDL-P: medium VLDL particles; S-VLDL-P: small VLDL particles; VS-VLDL-P: very small VLDL particles.
